# Decodable Spectral Structure of Input Noise in a Layered Cortical Spiking Network

**DOI:** 10.3390/brainsci16101117

**Published:** 2026-10-16

**Authors:** Mario Treviño, Oscar Arias-Carrión

**Affiliations:** 1 Laboratorio de Plasticidad Cortical y Aprendizaje Perceptual, Instituto de Neurociencias, Universidad de Guadalajara, Guadalajara 44130, Mexico; 2 División de Neurociencias Clínica, Instituto Nacional de Rehabilitación, Mexico City 14389, Mexico; 3 Tecnologico de Monterrey, Escuela de Medicina y Ciencias de la Salud, Mexico City 14380, Mexico

**Keywords:** colored noise, spectral exponent, recurrent cortical network, population decoding, laminar processing, asynchronous-irregular state, aperiodic exponent

## Abstract

**Highlights:**

**What are the main findings?**

- The spectral slope of input noise remained decodable from population activity.
- Decoding was highest in the stimulated layer-4 (L4) and second-highest in L2/3, and improved with noise contrast.

**What are the implications of the main findings?**

- Population statistics remain informative where single spikes cannot be traced.
- The network favored 1/*f*-like input, so some coding biases may derive from intrinsic dynamics.

**Abstract:**

Background/Objectives: Individual spikes are difficult to track through recurrent cortical circuits, where convergence, divergence, and nonlinear dynamics rapidly transform activity. However, the statistical properties of an input may remain detectable even when individual spike identity does not. We asked whether the spatiotemporal spectral slope of colored noise remained detectable as activity propagated through a simulated cortical network. Methods: We built a biologically inspired, four-layer recurrent spiking network based on mouse primary visual cortex. A drifting grating was embedded in colored noise spanning five spectral conditions (violet, blue, white, pink, and brown), while noise contrast and stimulus drive were varied independently. The network operated in an asynchronous-irregular regime, and population spectra were characterized using their aperiodic component. Results: Population activity changed systematically with the spectral slope of the noise rather than separating into discrete color representations. A continuous decoder recovered the spectral exponent *β* from population activity. Decoding generalized to new noise images the decoder had never seen. Removing all synaptic connections left L4 decoding unchanged, showing that the spectral signal there did not require recurrent interaction; recurrence instead determined how much of it reached the other layers. A feedforward model that received the same stimulus but lacked cellular dynamics did not support decoding. Decoding was highest in L4, whereas the univariate effect of *β* on firing rate peaked one stage downstream, in L2/3, and increasing noise contrast improved spectral discriminability. Recruiting feedforward inhibition in L4 did not change decoding accuracy. Conclusions: Recurrent cortical dynamics can transform input activity while retaining information about its statistical structure. This information was distributed across multiple dimensions of population activity and remained recoverable across cortical layers. Tracking population statistics rather than individual spikes provides a tractable framework for studying how recurrent circuits transform and retain input activity.

## 1. Introduction

Exploring how information propagates through neural circuits remains a central problem in computational neuroscience. One direct approach is to track individual spikes, or coordinated spike patterns, across successive neuronal populations. In recurrent cortical circuits, however, this quickly becomes difficult. Although the origin of an individual spike is unambiguous, its contribution to downstream activity is not. A single presynaptic spike can diverge onto many postsynaptic targets while convergent inputs, recurrent interactions, nonlinear membrane dynamics, and synaptic filtering reshape this activity at each stage. After only a few synaptic steps, it becomes difficult to determine which upstream neurons contributed to a given downstream spike and through which pathways.

A different property of neural activity may survive this transformation: its statistical structure. Rather than asking whether individual spikes can be followed through a circuit, we can ask whether a defined statistical property of an input remains detectable as activity propagates through successive processing stages. This question also changes how sensory noise can be viewed. If cortical circuits rapidly suppress externally imposed variability, then sensory noise should have little influence on downstream processing, and manipulating external noise would primarily influence early sensory stages. If, instead, the statistical properties of the input survive propagation or are systematically transformed by the circuit, external noise could serve to probe neural computation. In this view, noise is not simply a perturbation to be averaged away, but a measurable component of the input whose structure can be followed through the network.

To study this question quantitatively, the statistical property being tracked should be defined in advance and varied along a single, controlled dimension. Spectral structure provides such a parameter. Noise is characterized not only by its magnitude but also by how its power is distributed across frequencies. The power spectrum of many stochastic signals follows a power law,

S(*f*) ∝ 1/*f^β^* (1)

in which the spectral exponent *β* provides a continuous measure of noise color. Negative values shift power toward higher frequencies, whereas positive values shift power toward lower frequencies. The range examined here spans violet (*β* = −2), blue (*β* = −1), white (*β* = 0), pink (*β* = +1), and brown (*β* = +2) noise.

The spectral exponent also has a direct temporal interpretation. At *β* = 0, power is distributed equally across frequencies. More negative values produce faster fluctuations with weaker temporal correlations, whereas more positive values produce slower fluctuations with longer temporal correlations and larger excursions. *β*, therefore, provides an ordered and physically defined stimulus parameter: increasing *β* corresponds to progressively greater power at low frequencies. Because it varies continuously, its effects can be quantified along a single axis rather than treating different noise colors as unrelated stimulus categories. Moreover, this exponent applies to the full spatiotemporal stimulus, so the temporal spectrum reaching a single neuron is only a portion of that spectrum. We use *β* throughout as an ordered stimulus parameter, not as the temporal slope experienced by a single neuron.

Spectral structure is also relevant to natural vision. Natural images exhibit approximately 1/f^2^ spatial statistics, and natural movies show similar temporal scaling [1,2,3,4]. Eye and head movements further transform spatial structure into temporal fluctuations on the retina while partially whitening the input available to the visual system [5,6]. The spectral structure reaching the visual cortex is therefore shaped jointly by environmental statistics and by the observer’s movements. Spectral structure is consequently an ecologically relevant property of visual input rather than an arbitrary feature of stimulus design.

The statistical structure of natural stimuli has shaped theories of sensory processing and visual organization. Efficient coding theory proposes that sensory circuits are adapted to the statistical regularities of natural inputs, reducing redundant information while preserving informative structure [7,8]. Consistent with this view, coding in the fly retina matches the distribution of contrasts encountered in the natural environment [9], neurons in the cat lateral geniculate nucleus preferentially whiten natural movies while leaving white-noise stimuli largely unfiltered [10], and mouse V1 allocates its representational resources in a manner consistent with the statistics of natural images [2]. Although the underlying mechanisms differ across species and sensory stages, these findings support a common principle: early visual processing is sensitive to the statistical structure of sensory inputs.

Most of this evidence, however, involves individual neurons or relatively early sensory stages. What happens to such statistical structure within recurrent cortical circuits remains less clear. Does information about the spectral structure of an input remain detectable in population activity, or is it progressively lost as activity propagates through the network? Addressing this question experimentally is difficult because the statistical properties of natural scenes are strongly correlated. Luminance, contrast, spatial frequency, temporal correlations, chromatic content, and object identity vary together, making it difficult to attribute a neural response to any one of these properties.

We addressed this question using network simulations in a biologically inspired recurrent spiking network whose cellular, synaptic, and laminar organization was derived from cortical physiology. A simulated circuit allows stimulus properties to be controlled precisely while providing simultaneous access to the activity of every neuron. It also allows the relevant circuit mechanisms to be specified explicitly and the same network to be tested under every stimulus condition, so that changes in population activity can be attributed to the statistical structure of the input rather than to biological variability, electrode drift, or shifts in behavioral state. We therefore adopted the classical signal-plus-noise framework used in psychophysics [11,12]. A drifting grating with fixed contrast, orientation, and temporal frequency served as the deterministic signal, while the spectral slope of the superimposed noise was varied systematically along the *β* axis. The grating and total noise variance were held constant across conditions, so the manipulation changed the distribution of noise power across frequencies without changing its overall magnitude. This design isolated spectral structure while avoiding the multiple covarying stimulus dimensions characteristic of natural scenes.

Whether recurrent cortical networks retain information about the spectral structure of their inputs is not clear. Different properties of neural circuits lead to opposing predictions. For example, nonlinear threshold systems can use external noise to enhance signal transmission through stochastic resonance [13,14,15]. The effectiveness of this process depends on the temporal correlations of the noise, and colored noise can enhance weak signals more effectively than white noise under appropriate conditions [16,17,18]. If such mechanisms dominate, inputs that differ only in spectral slope should produce distinguishable population states.

Other properties of cortical circuits predict the opposite. Recurrent inhibition, variability quenching, and sub-additive response scaling can reduce trial-to-trial variability and decorrelate neural activity [19,20]. These mechanisms could progressively suppress externally imposed statistical structure as activity propagates through the network, leaving downstream populations responsive to the presence of noise but less sensitive to its spectral composition. Because these processes operate simultaneously and depend on the circuit’s specific properties, their combined effect is hard to determine in biological systems. Simulation therefore provides a direct way to test whether information about spectral structure remains detectable as activity propagates through recurrent cortex.

We asked three questions. Does population activity depend on the spectral properties of the input, or only on the presence of noise? Can *β* be recovered from population activity, and does this generalize to a new noise field? Is *β* represented uniformly across the cortical hierarchy, or is it concentrated in particular layers? We expected spectral structure to emerge as a graded property of population activity, while network dynamics would determine its strength and laminar distribution.

## 2. Materials and Methods

### 2.1. Overview

We simulated a multilayer recurrent spiking network composed of biophysically inspired Hodgkin–Huxley-type neurons. We presented drifting gratings combined linearly with colored noise to the input layer, and recorded network activity for offline analysis of how recurrent processing transformed the stimulus’s statistical structure. The following sections describe the network architecture, neuronal and synaptic models, connectivity, background activity, stimulus generation, simulation procedures, two control experiments, and statistical analyses, in the order they entered the simulation pipeline.

The network incorporated several structural and physiological features of neocortical circuits, including biophysical models of pyramidal and fast-spiking neurons, dual-exponential synapses, distance-dependent connectivity, a four-layer cortical organization with recurrent feedforward and feedback projections, physiological axonal delays, and ongoing background activity. We based anatomical parameters on measurements from the mouse primary visual cortex (V1) whenever available. The model was not intended to reproduce the full complexity of the cortical microcircuit. Instead, it provided a controlled framework for testing whether the statistical structure of sensory inputs remained detectable in recurrent population activity. We therefore simplified several features, including using a single inhibitory interneuron class, current-based synaptic interactions, simplified external stimulation, and a single fixed connectivity generated once and reused for every simulation. We describe these simplifications below, along with their implications for interpreting the results.

We performed all simulations using a layered, distance-dependent cortical network simulator written in Python 3.11. Simulation outputs were exported as HDF5 files and reduced to per-simulation feature vectors using custom Python 3.11 scripts with NumPy and h5py. We performed inferential statistics and decoding analyses in MATLAB R2023b using the Statistics and Machine Learning Toolbox. We stored simulation parameters, connectivity matrices, synaptic weights, conduction delays, and random seeds with each simulation. All analyzed simulations used a single network: we generated neuron placement, connectivity, synaptic weights, and conduction delays once and reused them in every run. Within that fixed network, the per-cell tonic currents and the Ornstein–Uhlenbeck background activity (see below) were drawn afresh for each simulation from fixed distributions, so background activity varied from run to run while network structure did not. Fixing the network isolated stimulus- and background-driven effects from wiring variability; drawing the background per run means a decodable stimulus effect is not tied to a single background trajectory.

### 2.2. Column Geometry

The network consisted of four concentric spherical shells representing cortical layers L2/3, L4, L5, and L6. Shell radii were 50, 70, 89, and 109 µm, respectively. Each shell carried the same number of neurons, so surface density fell with shell radius, from 12,700 cells/mm^2^ in L2/3 to 2700 cells/mm^2^ in L6. Layers therefore differed in local neuronal packing, and this difference could contribute to laminar differences in the results; the ablation experiment below separates this contribution from synaptic interaction.

We distributed neurons on each shell using a spherical Fibonacci, or golden-angle, lattice [1]. This arrangement provided approximately uniform nearest-neighbor spacing without introducing preferred spatial directions and was exactly reproducible for a given number of neurons. Within each layer, we calculated intersomatic distances as great-circle distances along the corresponding spherical surface. For interlaminar connections, we projected source neurons radially onto the target shell before calculating the corresponding distance, maintaining topographic alignment between cortical layers.

The production network contained 1600 neurons: 1280 excitatory pyramidal neurons (320 per layer) and 320 inhibitory fast-spiking interneurons (80 per layer), corresponding to an 80:20 excitatory-to-inhibitory ratio within each layer. Neurons were stored in layer-blocked order, with the 320 pyramidal cells of a layer preceding its 80 interneurons; layer membership for all analyses was read from the stored network file.

### 2.3. Neuronal Models

We simulated excitatory neurons using the two-compartment Pinsky–Rinzel reduction in the Traub pyramidal-cell model [2]. The somatic compartment contained fast sodium and delayed-rectifier potassium conductances responsible for action-potential generation, whereas the dendritic compartment included persistent calcium, calcium-dependent potassium, and afterhyperpolarization currents. The two compartments were coupled through an axial conductance, resulting in a model with nine dynamic state variables. We selected this model because it retains dendritic calcium-dependent bursting and the associated slow conductances that shape the neuron’s low-frequency dynamics. These properties were particularly relevant here because the stimulus manipulation was defined by its temporal spectral structure.

We simulated inhibitory neurons using the single-compartment Wang–Buzsáki model [3], which captures the fast-spiking behavior characteristic of cortical inhibitory interneurons. We used a single fast-spiking population to represent the inhibitory population in each layer. We did not model the full diversity of cortical interneurons, which includes several molecular and functional subclasses, such as PV-, SST-, and VIP-expressing cells [4]. This simplification restricts the analysis to fast-spiking, predominantly perisomatic inhibition and does not address dendrite-targeting or disinhibitory inhibitory motifs.

We integrated state variables using the exponential Euler method, precomputing voltage-dependent transition rates in lookup tables during simulator initialization. Unless otherwise stated, neuronal parameters followed the original published formulations. Pyramidal neurons used the parameterization of Pinsky and Rinzel [2], and fast-spiking interneurons used the original Wang–Buzsáki parameterization [3].

Within each cell class, all model neurons were biophysically identical, with the same channel densities, kinetics, and compartmental geometry. We introduced heterogeneity at the operating-point level. Each cell, pyramidal and fast-spiking alike, received an independent tonic depolarizing current drawn from a class-specific distribution and held constant throughout the simulation. The mean and standard deviation were 1.379 ± 0.039 µA·cm^−2^ for pyramidal somata and 0.227 ± 0.108 µA·cm^−2^ for interneurons. We fixed these distributions, but redrew the per-cell values for every simulation. Cells therefore occupied different distances from threshold and converted the same synaptic input into different firing rates. This dispersion, rather than differences in underlying conductances, explains the observed spread in spontaneous firing rate among pyramidal neurons (0.5–5 Hz).

This was a deliberate modeling choice. The model does not reproduce the full biophysical diversity of cortical neurons, including differences in channel complements, morphology, or intrinsic excitability. Instead, we introduced variability in neuronal activity while keeping the underlying biophysical mechanisms uniform. The resulting population therefore captures functional heterogeneity in firing rate and excitability without introducing additional mechanistic differences between cells. This lets us interpret differences across layers, cell types, or stimulus conditions without attributing them to unmodeled biophysical heterogeneity.

### 2.4. Synaptic Transmission

We modeled synaptic interactions using dual-exponential conductance kernels with separate rise and decay time constants. Postsynaptic currents were calculated from the synaptic conductance and the difference between membrane potential and the corresponding reversal potential:

(2) $$I_{syn}=g_{syn}\left(t\right)\left(V-E_{syn}\right)$$

where $E_{syn}=0$ mV for AMPA-mediated excitation and −75 mV for GABA-mediated inhibition. We precomputed synaptic kernels for each synapse class and truncated them once the conductance decayed below 0.1% of its peak value. Precomputation avoided repeatedly evaluating identical synaptic waveforms during simulations and reduced computational cost. The simulator also included an optional NMDA conductance implemented as a dual-exponential synapse with voltage-dependent magnesium block [5]. NMDA conductances were disabled in all simulations reported here. Recurrent excitation therefore operated through AMPA-like synaptic currents with relatively rapid decay rather than through a slower NMDA-mediated component. For computational efficiency, we approximated synaptic interactions as synaptic currents throughout the simulations. This approximation preserves the temporal dynamics of synaptic excitation and inhibition while avoiding the additional state-dependent conductance effects of a fully conductance-based implementation.

### 2.5. Connectivity

Synaptic connectivity was generated independently for each ordered source–target cell population pair using a distance-dependent Bernoulli process. The probability of a connection from presynaptic neuron i to postsynaptic neuron j was defined as

(3) $$P_{ij}=p_{max}exp\left(-\frac{d_{ij}^{2}}{2{\left(\sigma R_{tgt}\right)}^{2}}\right)$$

where $d_{ij}$ is the arc-length distance between the two neurons, $R_{tgt}$ is the radius of the target layer, $p_{max}$ is the maximum connection probability, and σ defines the spatial spread relative to the target-layer radius. Expressing the spatial spread in units of the target-layer radius kept the connectivity profile shape comparable across layers of different size.

Within each cortical layer, we implemented four canonical connection classes: pyramidal-to-pyramidal (PY → PY), pyramidal-to-interneuron (PY → INT), interneuron-to-pyramidal (INT → PY), and interneuron-to-interneuron (INT → INT). Interlaminar projections consisted of the principal excitatory feedforward pathway (L4 → L2/3 → L5 → L6) and a feedback projection from L6 to L4. Maximum connection probabilities and spatial spreads were selected within experimentally reported ranges for mouse neocortex [6,7,8,9,10].

The strongest interlaminar projection was L4 → L2/3 ($p_{max}=0.35$). Successive feedforward projections used progressively lower connection probabilities. The L2/3 → L5 projection had a broader spatial spread (σ=0.7R), reflecting the extended arborization of layer-2/3 pyramidal neurons onto layer-5 apical dendrites [11]. The L6 → L4 feedback projection was weak and spatially broad ($p_{max}=0.08$, σ=0.60), consistent with the broad modulatory organization of corticothalamic feedback circuits [12,13,14]. Cross-layer inhibitory projections remained sparse ($p_{max}\approx 0.02$, σ=0.30), reflecting the predominantly intralaminar organization of cortical interneuron axons [15,16].

The resulting network contained 96,760 synaptic connections distributed across 29 projection ‘pathways’. Connection probability decreased exponentially with intersomatic distance, with a characteristic spatial decay of 261 µm. The mean synaptic weight was 0.34, and neurons formed an average of 60.5 outgoing connections. All analyzed simulations used the same fixed network realization, so we could not attribute differences between stimulus conditions to differences in network wiring. Therefore, this analysis does not assess variability across independently generated network realizations, which remains an important direction for future work.

### 2.6. Axonal Delays

We assigned conduction delays independently to each synaptic connection as the sum of three components: a fixed synaptic delay, a distance-dependent conduction delay, and a layer-dependent translaminar offset. The fixed synaptic delay was 0.5 ms. We calculated the lateral conduction component from the chord distance between neurons using a conduction velocity of 0.3 m/s, consistent with measurements from unmyelinated intracortical axons [17,18].

Because the concentric spherical shells represent cortical layers without explicitly modeling their radial separation, we added a layer-specific offset to account for the anatomical distance between laminae. We derived these offsets from atlas-based estimates of mouse V1 layer depths [19], ranging from 0.667 ms between adjacent layers to 2.167 ms between L2/3 and L6. We capped total conduction delays at 5 ms, which remained within experimentally reported ranges for intracortical synaptic transmission [6,20,21]. Across the network, delays averaged 0.80 ms (range: 0.53–1.64 ms, within-layer pathways).

These delays influenced the timing of synaptic interactions and therefore the temporal structure of population activity. However, they were short relative to the 1 s analysis window and, together with the short AMPA and GABA synaptic time constants, did not impose a fixed low-frequency timescale on the population response. This distinction matters because the analysis focused on whether the network response reflected the spectral structure of the stimulus rather than on a low-frequency rhythm imposed by the conduction delays.

### 2.7. Background Activity

To maintain ongoing activity in a regime representative of awake cortex, each neuronal compartment received an independent Ornstein–Uhlenbeck stochastic current [22,23]. The process was defined by

(4) $$\frac{dI}{dt}=-\frac{I-I_{mean}}{\tau }+\sigma \sqrt{\frac{2}{T}}\xi \left(t\right)$$

where τ is the correlation time, σ is the noise amplitude, and $\xi_{t}$ is Gaussian white noise, and *I*_mean_ is the per-cell tonic depolarization described above. The process was integrated using its exact discrete-time solution rather than the Euler–Maruyama approximation. With the 0.025 ms integration timestep and 5 ms correlation time used here, this approach preserved the stationary variance of the stochastic process without the discretization bias that can arise from Euler–Maruyama integration.

We fixed the correlation time at 5 ms. Noise amplitudes were assigned according to neuronal class and compartment, with pyramidal somata receiving σ=2.70 µA/cm^2^ and interneurons receiving σ=1.26 µA/cm^2^. These parameters kept pyramidal neurons about 7–10 mV below the firing threshold while producing membrane-potential fluctuations of about 3 mV, consistent with measurements from the awake cortex [24]. Spontaneous pyramidal firing rates stabilized between 0.5 and 5 Hz, reflecting per-cell dispersion in Imean rather than differences in intrinsic conductances.

The background current was independent across compartments. It therefore provided ongoing intrinsic variability without introducing a shared temporal structure across neurons that could be mistaken for a stimulus-driven effect. This background activity placed the network in an asynchronous, irregular operating regime, commonly observed in recurrent cortical network models [25,26]. Maintaining this ongoing activity also prevented the stimulus from being evaluated against an otherwise quiescent network and provided a background state through which feedforward inputs could propagate [27].

The present implementation used a current-based approximation of the conductance-driven background activity described by Destexhe and Paré [23]. Thus, it reproduced membrane-potential fluctuations and spontaneous firing statistics associated with a high-conductance-like regime, but it did not explicitly reproduce the reduction in input resistance produced by fluctuating synaptic conductances. This is a limitation: the background drive was intended to reproduce the dynamical operating regime relevant to the present analysis, rather than to provide a detailed biophysical reconstruction of synaptic bombardment.

### 2.8. Visual Stimulation

We delivered visual stimuli exclusively to layer-4 (L4) neurons, which correspond to the model’s primary feedforward input layer. In mouse V1, thalamic afferents from the dorsal lateral geniculate nucleus predominantly target excitatory and fast-spiking neurons in L4, while activity reaches superficial and deep cortical layers through intracortical connections [28,29]. We therefore restricted external stimulation to L4, following the general organization used in previous biophysical models of mouse V1 [30].

This arrangement was also important for the laminar analysis. Because L2/3, L5, and L6 did not receive the stimulus directly, any stimulus-dependent spectral signature detected in these layers had to emerge through intracortical processing rather than from direct external stimulation.

We presented stimuli as spatiotemporal image sequences. Each L4 neuron sampled the stimulus by orthographically projecting it onto the image plane, then applying bilinear interpolation. Samples falling outside the stimulus boundaries were clamped to the nearest valid pixel. We computed the projection geometry, including the mapping from each neuron’s position on the L4 shell to an image-plane coordinate, once during initialization and held it fixed throughout the simulation. The mapping did not move, rotate, or otherwise change over time; we updated only the pixel value sampled at each fixed coordinate for each stimulus frame.

Stimulus frames were presented at 60 Hz (16.667 ms per frame), matching the refresh rate used in our psychophysical experiments. This choice provides a common temporal sampling rate between the simulated and human experiments, allowing direct comparison of stimulus timing. We integrated neuronal dynamics at a much finer timestep of 0.025 ms to resolve the fast membrane and synaptic dynamics of the neuronal models. The integration timestep was independent of the stimulus presentation rate. The external drive was:

I_ext_ = g · S_bilinear_ (5)

where g is the stimulus gain in µA/cm^2^. We refer to g  throughout the manuscript as the stimulus drive strength. Gain scaled only the injected current amplitude and did not alter the stimulus’s spatial or spectral structure. Drive strength and spectral slope were therefore independent experimental variables, allowing us to evaluate the effect of β separately across different network operating points.

Stimulus sampling therefore followed a two-rate scheme. We recomputed the interpolated pixel value for each L4 neuron once per 16.667 ms frame and held it constant throughout that frame, producing a zero-order-hold stimulus waveform updated at 60 Hz. The held value was scaled by the gain *g* (Equation (5)) and added to the membrane equation at every 0.025 ms integration step, with one stimulus value applied to each neuron at each timestep. The resulting external drive was an instantaneous current offset rather than a synaptic event: it had no rise time, decay, or biphasic waveform and was added directly to the membrane equation without convolution with a synaptic kernel. Thus, we delivered the stimulus as a current clamp rather than through a simulated synapse.

We implemented external visual input as a deterministic current rather than explicit thalamocortical spike trains. Under the diffusion approximation, synaptic input produced by presynaptic spiking can be represented by an equivalent mean current with stochastic fluctuations [31]. This implementation allowed independent manipulation of the external stimulus amplitude, separate from additional presynaptic parameters. We did not apply receptive-field kernels, such as Gabor or difference-of-Gaussians filters. Instead, neurons sampled the image directly. This preserved the stimulus’s spectral statistics and avoided imposing an additional receptive-field transformation at the network input. Consequently, the spectral structure delivered to L4 matched the stimulus specification rather than the output of an assumed visual preprocessing stage.

### 2.9. Colored-Noise Generation

The experimental manipulation varied the spectral slope of the stimulus noise while keeping its overall variance constant. We generated noise using the colored noise implementation [32]. Briefly, we generated isotropic Gaussian white noise in three dimensions (two spatial and one temporal), transformed it to the Fourier domain, multiplied it by a radially symmetric power-law filter, and transformed it back to the spatial domain using an inverse fast Fourier transform. The filter was defined as

|F(*f*)| ∝ *f*^−(*β*+*d*−1)/2^ (6)

where d=3 denotes the dimensionality of the spatiotemporal stimulus and *f* is the radial frequency in the three-dimensional Fourier volume. The generator was parameterized by an internal exponent α, which relates to the conventional noise-color parameter used throughout this paper by:

(7) β=−α

so that increasing β corresponded to progressively greater power at lower frequencies. The five stimulus conditions therefore corresponded to violet (β=−2), blue (β=−1), white (β=0), pink (β=+1), and brown (β=+2) noise. For clarity, each noise field corresponds to one specific random draw of colored noise: a single three-dimensional array of values with the intended spectral slope. Each color was represented by one such field, and its contrast levels were scaled copies of it. Two spectral conventions should be distinguished. The exponent in Equation (1) describes the total power across all modes at a given radial frequency. The power of an individual mode follows a different exponent because the number of modes increases with frequency. This distinction is important for interpreting Equation (6), particularly for the violet condition.

Because we filtered jointly across the full three-dimensional Fourier volume, the power law controlled both spatial and temporal frequencies. The resulting stimuli therefore defined spatiotemporal statistics rather than independently generated spatial noise in each frame. Frame-by-frame spatial noise would have produced a temporally white sequence regardless of the nominal spatial color and would therefore not have provided the intended manipulation of temporal spectral structure. The temporal spectrum reaching a single L4 neuron is obtained by sampling the full spatiotemporal stimulus along one spatial location. It therefore does not directly carry the exponent *β*. Its temporal slope changes systematically with *β* but is compressed relative to the imposed exponent. We therefore use *β* throughout as an ordered stimulus parameter, not as the temporal slope of the input to any single neuron.

We imposed the spectral exponent analytically during stimulus construction, so it was known exactly for every stimulus. We used spectral estimates calculated from the rendered stimuli only to verify the filtering procedure; we did not use them to define the independent variable.

### 2.10. Stimulus Construction

The visual signal consisted of a drifting sinusoidal grating generated on the same spatiotemporal grid as the noise field. Spatial frequency was fixed at four cycles across the image, orientation was held constant at a single tilt in degrees, and temporal drift was identical across all conditions. The grating was therefore the same across all stimulus conditions and could not account for differences between noise colors.

Signal contrast was defined using Michelson contrast,

(8) $$C_{signal}=\frac{I_{max}-I_{min}}{I_{max}+I_{min}}$$

and the grating contrast was fixed at $C_{signal}=0.125$. We normalized each generated colored-noise field to unit standard deviation before combining it with the grating. This normalization ensured that the different spectral conditions had the same total noise variance while retaining their differences in the distribution of power across frequencies. Without this normalization, low-frequency-dominated noise would also differ in total power, making it difficult to distinguish the effect of spectral slope from that of noise magnitude.

The final stimulus was defined as

(9) $$S_{total}=w_{signal}S+w_{noise}N$$

where S denotes the drifting grating, N the normalized colored-noise field, and $w_{signal}$ and $w_{noise}$ their respective weights. We added the two components pixel by pixel and frame by frame. Thus, when we refer to a grating embedded in colored noise, we mean an additive combination of two independent stimulus components. We could therefore vary the grating orientation and the noise’s spectral exponent independently, without multiplicative contrast modulation, masking, or occlusion between the two components.

We fixed the signal weight at the value corresponding to 12.5% Michelson contrast, a contrast commonly used in signal-in-noise psychophysical experiments [33,34,35]. We varied noise contrast across nine non-zero levels, along with a zero-noise condition. The latter contained the drifting grating alone and served as an internal control.

We varied stimulus drive strength across five values (2.5, 5, 10, 20, and 40 µA/cm^2^). The effective per-neuron drive delivered to layer 4 (L4), measured after normalization and bilinear sampling at the cell locations, rose modestly and monotonically across the color series (mean absolute current 6.96 µA/cm^2^ for violet to 7.77 for brown, an 11.6% spread, *r* = 0.98 with *β*). This spread arises from the sampling geometry rather than from an error in stimulus construction. Although we normalized each noise field to unit variance, L4 neurons sampled the field via bilinear interpolation, which acts as a mild low-pass filter. Low-frequency-dominated fields are less affected by this averaging, so their sampled variance increases with *β*. Thus, with finite spatial sampling, we cannot maintain both unit variance of the full stimulus and equal variance of the input delivered to every neuron. We therefore retained this amplitude difference and tested whether it contributed to decoding using a feedforward model without cellular or network dynamics. Simulations were performed under two stimulation conditions: (1) stimulation of L4 pyramidal neurons only, and (2) simultaneous stimulation of L4 pyramidal neurons and fast-spiking interneurons. A single L4 bypass reference run, in which no drive was delivered to L4, was performed per gain. It carried no color, contrast, or stimulation condition. The second condition modeled feedforward inhibition from thalamocortical input to inhibitory neurons.

### 2.11. Stimulus Timing

Each stimulus sequence contained 64 frames presented at 60 Hz, yielding a duration of 1.067 s before repetition. The 60 Hz presentation rate matched the visual stimulus’s temporal sampling and the refresh rate of our psychophysical displays (see above), whereas we integrated neuronal dynamics at the much finer 0.025 ms timestep described above.

Because the stimulus sequence repeated every 1.067 s, its imposed temporal spectrum was defined only up to the Nyquist frequency of 30 Hz. Population activity was analyzed in four consecutive 1 s windows beginning 1 s after stimulus onset. We discarded the first second to exclude the initial transient and restrict the analysis to the steady-state response.

Each 1 s analysis window approximately matched one stimulus repetition. The repeated sequence therefore introduced a periodic component at approximately 0.94 Hz, corresponding to the 1.067 s stimulus loop. We did not remove this component from the population activity because the analysis characterized the resulting aperiodic component of the network spectrum rather than attempting to reconstruct the stimulus spectrum directly.

### 2.12. Numerical Integration and Parameter Tuning

We integrated network dynamics using a vectorized exponential Euler solver with a fixed timestep of 0.025 ms. Synaptic events were accumulated using precomputed dual-exponential kernels and vectorized updates, avoiding explicit iteration over individual neurons during the simulation. We recorded membrane potentials and spike times at the integration timestep, corresponding to a 40 kHz sampling rate, and did not temporally downsample them before analysis. Retaining the native temporal resolution avoided additional filtering or aliasing that temporal decimation would otherwise introduce, particularly for subsequent estimation of population spectra.

Model parameters were optimized in two stages using the multi-objective genetic algorithm NSGA-II implemented in pymoo [36,37]. We used a multi-objective approach because the network’s target properties could not be reduced to a single measure: spontaneous firing rate, spike-train irregularity, and population synchrony had to be matched simultaneously, and improving one property could move another away from its target.

In Phase 1, we ran simulations without external stimulation to establish a spontaneous activity regime representative of awake mouse V1. Optimization targets included pyramidal-cell firing rates of 3–8 Hz, interneuron firing rates of approximately 20 Hz, inter-spike interval variability ($CV_{ISI}\approx 1$), and low population synchrony (χ<0.10), consistent with experimental measurements [38].

In Phase 2, we used the best parameter sets from Phase 1 as the starting population and adjusted interlaminar synaptic gains to improve stimulus propagation. We constrained background-noise parameters to remain within ±30% of their Phase 1 optimized values. This constraint ensured that optimization for stimulus propagation did not substantially alter the spontaneous operating regime established during Phase 1.

We implemented short-term synaptic plasticity [39] and spike-timing-dependent plasticity [40] in the simulator but disabled them throughout the simulations reported here. Connectivity and synaptic parameters were therefore fixed across all stimulus conditions.

### 2.13. Data Reduction and Feature Extraction

The main experimental design comprised 505 simulations; two additional simulation sets, described below, tested generalization across noise fields (898 simulations) and the contribution of synaptic interaction (two ablated networks). We read simulation metadata from the configuration stored in each HDF5 output file.

The design crossed noise color (5 levels: violet, blue, white, pink, brown, corresponding to *β* = −2 to +2), noise contrast (10 levels: a grating-only level carrying no noise, plus nine levels rising to 12.5% Michelson contrast), and stimulation condition (2 levels), at five stimulus gains. Each gain block therefore contained 5 × 10 × 2 = 100 stimulated simulations, plus one layer-4 bypass reference run, giving 101 runs per gain and 505 in total. We ran the grating-only level once per color rather than sharing it across colors; because those runs contain no noise, they are identical across colors and collapse to a single distinct simulation per stimulation condition. At the analysis gain of 10 µA/cm^2^, this leaves 46 distinct simulations per stimulation condition (45 noise levels plus one grating-only run), 92 in total.

We reduced each simulation to one feature vector per cortical layer and neuronal population. Features were calculated separately for the four 1 s analysis windows described above and then averaged across windows, so that the simulation, not the window, was the unit of analysis. Thirteen features were computed: firing rate, coefficient of variation in the inter-spike interval, Fano factor, population synchrony (χ), aperiodic spectral exponent, local-field-potential standard deviation, mean membrane potential, membrane-potential standard deviation, and band-limited spectral power in five bands—delta (1–4 Hz), theta (4–8 Hz), alpha (8–13 Hz), beta (13–30 Hz), and gamma (30–80 Hz) [41,42]. The decoder excluded mean membrane potential and therefore operated on twelve features. Band names are spelled out throughout to avoid confusion with the spectral exponent *β* of Equation (1).

We selected the feature set to characterize population responses from complementary perspectives, including firing rate, spike-train variability, membrane-potential dynamics, synchrony, and spectral structure. The analyses, therefore, did not depend on a single class of response measure. We calculated cell-specific measures, including firing rate, coefficient of variation, and Fano factor, for individual neurons and then averaged them within each population. We calculated population measures, including synchrony, local-field-potential variance, spectral power, and the aperiodic exponent, directly from pooled population activity. Counts of distinct simulations are reported after this step, and a guard confirmed that no duplicated simulation contributed to both the training and the test partition of any cross-validation fold.

### 2.14. Spectral Analysis

We estimated power spectra from the native 40 kHz recordings as Hann-tapered periodograms. Each 1 s analysis window (40,000 samples) was tapered with a single 40,000-point Hann window and transformed with a 40,000-point discrete Fourier transform, giving a frequency resolution of 1 Hz and an equivalent noise bandwidth of 1.5 Hz. We computed one spectrum per analysis window; the four simulation windows did not overlap, and we averaged their spectra before further analysis. Because each segment spanned the full analysis window, we applied no within-window segmentation or overlap averaging, and the estimator is a Hann-tapered periodogram averaged over four segments rather than a within-window Welch estimate.

Because network activity remained in an asynchronous-irregular regime without dominant oscillatory peaks, we characterized the spectrum’s broadband, aperiodic component rather than focusing on individual oscillatory peaks [26,43]. We estimated the aperiodic exponent by ordinary least-squares regression of log-transformed power against log-transformed frequency over the 1–100 Hz range. We specified this frequency interval a priori and applied it identically across all experimental conditions. Because the frequency bins are linearly spaced, an ordinary least-squares fit in log–log coordinates weights more the high-frequencies. We applied the same fit to every condition, so this shifts the absolute exponent but not the comparisons between conditions.

The input spectral exponent and the network aperiodic exponent should be distinguished. We defined the imposed stimulus spectrum only below the 30 Hz Nyquist frequency of the 60 Hz stimulus sequence. The 1–100 Hz range used to characterize population activity therefore extended beyond the frequency range in which *β* was directly imposed at the input. The measured aperiodic exponent therefore describes the spectral organization of stimulus-driven network activity; it is not intended as a direct estimate or reconstruction of the input *β*.

We also evaluated a broader frequency range (1–10,000 Hz), which yielded greater separation between stimulus conditions. We did not use this range for the primary analyses because estimates of the aperiodic exponent depend on the selected frequency interval [44]. Selecting the interval that maximizes the separation between conditions would introduce a data-dependent choice of the analysis range. We therefore retained the narrower 1–100 Hz interval defined a priori.

### 2.15. Generalization Across Independent Noise Fields

The main design used one noise field per color, with contrast levels generated by scaling that field. To test generalization, we generated ten independent fields for each color. We produced each field with the same spectral filter, volume, and normalization as the main stimuli, and simulated it at a gain of 10 µA/cm^2^ under both stimulation conditions across all contrast levels. We trained the decoder on nine fields and tested it on the tenth, so no field appeared in both the training and test sets. We repeated the within-field analysis on the same simulations using the same features and classifier.

### 2.16. Ablation and Null Networks

We used two ablated networks to determine whether color decoding required synaptic interactions. All neuronal, stimulus, and analysis parameters matched those of the intact network; only the synaptic weights were changed. In the disconnected network, we set all synaptic weights and interneuron gap junctions to zero. In the no-intralaminar network, within-layer connections and gap junctions were removed while interlaminar projections were retained. These two conditions separate the contribution of local recurrence from feedforward transmission. Both retain the full Pinsky–Rinzel and Wang–Buzsáki biophysics, the per-cell tonic currents, and the background drive. Neither therefore tests whether the stimulus itself is sufficient for decoding.

A third control addresses this question. This feedforward model used the same stimulus delivery pipeline but omitted network and cellular biophysics. The stimuli were sampled at the L4 cell locations using the same projection and bilinear interpolation as in the full model. These inputs then drove single-compartment leaky integrate-and-fire units with the same membrane time constant and threshold, but without synaptic connections. The model therefore preserved the stimulus, membrane filtering, and spike threshold while removing the full network’s intrinsic and recurrent dynamics. We then applied the same features and classifier.

### 2.17. Statistics

We treated each simulation as the unit of analysis. For each simulation, we averaged the four 1 s analysis windows before statistical testing, so repeated windows from the same simulation did not count as independent observations. The zero-noise condition contained no colored noise and served as an internal negative control. We therefore excluded it from analyses of spectral slope, leaving the nine non-zero noise levels for regression and decoding analyses.

The primary analysis tested whether population responses varied systematically with the stimulus’s spectral exponent. Noise colors were therefore treated as an ordered continuous variable,

*β* = −2, −1, 0, +1, +2,

corresponding to violet, blue, white, pink, and brown noise, respectively, rather than as five unordered categories. This approach tested the specific hypothesis that responses changed monotonically along the spectral axis, thereby reducing the analysis to a single degree of freedom.

For each combination of cortical layer, cell type, stimulus drive, and stimulation condition, we regressed population metrics on *β* using ordinary least-squares regression. We report regression slopes as the change in the response variable per one-unit increase in *β*, along with 95% confidence intervals. Partial $\eta^{2}$ was used as the effect-size measure.

To determine whether a linear model adequately described the relationship between response magnitude and β, we also compared models with linear and quadratic *β* terms. We retained the quadratic term only if it provided evidence of a systematic departure from linearity after correcting for multiple comparisons.

The comparison between the two stimulation conditions was made with a linear mixed-effects model of firing rate on the fixed effects of spectral slope, stimulation condition, and their interaction, with random intercepts and random slopes on *β* by run. This model was used rather than a second set of separate regressions because the two stimulation conditions were run over matched design cells and the question concerns their interaction with *β* rather than their marginal means.

Multiple comparisons were controlled using the Benjamini–Hochberg false-discovery-rate procedure within predefined families of tests [45]. Adjusted probabilities are reported as q-values. We defined families as the set of layer × cell-type tests for a given metric. Because tonic currents and background activity were drawn independently for each run within one fixed network, the tests generalize over stimuli and over background activity within that network, but not over networks. The inferential unit is the simulation, and *n* is the number of distinct simulations after removing byte-identical duplicate runs. Wilson 95% confidence intervals are reported for all accuracies, and significance was assessed against permutation nulls rather than theoretical chance [46]. The continuous *β* decoder was ordinary least-squares regression on the same z-scored features and the same folds, reported as the correlation between predicted and true *β* and the RMSE in *β* units. We additionally evaluated the robustness of the principal results using the more conservative Benjamini–Yekutieli procedure, which remains valid under arbitrary dependence among tests [47].

The strength of the color-specific population signature was quantified as the standardized absolute deviation of each color mean from the grand mean. Confidence intervals were obtained using percentile bootstrap resampling with 5000 resamples. Stability of the ordering of the five noise colors across stimulus gains was quantified using Kendall’s $\tau_{b}$.

We performed population decoding using a five-class nearest-centroid classifier trained on 12 z-scored response features; we excluded mean membrane potential from the classifier. We selected a nearest-centroid classifier because it does not require estimating model hyperparameters and therefore avoids an additional model-selection step. It provides a simple and conservative test of whether the spectral condition is available in the multivariate population response.

We tested the decoder by leaving out one noise contrast level at a time. The nine non-zero levels give nine folds: in each, the classifier learns the five colors from eight levels and is then asked to label the simulations at the ninth, which it has never seen. Performance therefore reflects whether a color can be identified at a contrast the decoder was not trained on. Within each fold, the mean and standard deviation used to z-score the features were computed on the training levels alone and applied unchanged to the held-out level, so nothing about the test condition enters the feature scaling.

We assessed classification significance using 10,000 label permutations while preserving the cross-validation structure. We calculated empirical *p*-values from the resulting permutation distributions and corrected for multiple comparisons using the same Benjamini–Hochberg procedure described above. Per-class sensitivity was calculated from the diagonal elements of the pooled confusion matrix. Because the five-way accuracy point estimate is sensitive to the fold assignment at this sample size, accuracy is reported as Wilson 95% confidence intervals and the random-number generator seed used for fold assignment was fixed.

Stimulus gains producing less than a 10% increase in L4 pyramidal firing relative to the unstimulated reference were considered below the effective response threshold. The two lowest gains (2.5 and 5 µA/cm^2^) fell below this threshold; we retained them as subthreshold controls but did not give them inferential weight. Unless a gain is stated explicitly, decoding results are reported at 10 µA/cm^2^, and univariate effect sizes at 40 µA/cm^2^. All simulations were performed in Python 3.11 using NumPy, h5py, and pymoo. Statistical analyses were conducted in MATLAB R2023b using the Statistics and Machine Learning Toolbox.

## 3. Results

### 3.1. Pyramidal and Interneuron Models Have Distinct Input–Output Functions

Before examining network activity, we characterized the firing properties of the two neuronal models used in the simulations. The pyramidal and fast-spiking interneuron models responded differently to depolarizing current steps (Figure 1a–d). The two-compartment Pinsky–Rinzel pyramidal neuron generated bursts in which dendritic calcium spikes prolonged depolarization and reinitiated somatic sodium spikes (Figure 1a,d). In contrast, the single-compartment Wang–Buzsáki interneuron produced sustained, non-adapting tonic firing, consistent with a fast-spiking phenotype (Figure 1b).

Both models had similar rheobase (0.2 µA/cm^2^), but their firing rates increased at different rates above threshold. The f–I slope was 9.26 Hz per µA/cm^2^ for pyramidal neurons and 20.48 Hz per µA/cm^2^ for interneurons, corresponding to a 2.2-fold higher gain in the interneuron model (Figure 1c,f). The two cell types had comparable firing thresholds but distinct input–output functions. These f–I curves also set the scale for the tonic depolarizing currents used to impose functional variability across the population (Equation (4), *I*_mean_; see Section 2). Varying this magnitude (a fraction of a µA/cm^2^ across cells) is enough to produce the several-hertz range of spontaneous rates observed in the assembled network.

The intrinsic spike waveform of the pyramidal model was also maintained during network activity. The mean pyramidal-cell spike waveform recorded in the recurrent network was nearly identical to that measured in isolation (Figure 1e). Recurrent synaptic input therefore altered firing rate and spike timing without substantially changing the shape of the action potential.

### 3.2. The Network Reproduces Laminar and Distance-Dependent Connectivity

We next characterized the network architecture before examining how activity propagated through it. We distributed the 1600 neurons across four concentric shells representing cortical layers L2/3, L4, L5, and L6, using a spherical Fibonacci lattice that provided approximately uniform spacing within each layer (Figure 2a). Synaptic connectivity was strongly distance-dependent, following the Gaussian probability rule of Equation (3): connection probability decreased with increasing intersomatic distance across the four within-layer connection classes (PY → PY, PY → INT, INT → PY, and INT → INT; Figure 2b,c). An exponential function described the distance dependence, with a characteristic decay length of 261 µm (1/e distance). Connectivity remained predominantly local across both excitatory and inhibitory populations rather than being determined primarily by cell type or layer membership.

The network also showed the intended laminar organization of excitatory and inhibitory projections (Figure 2d,e). Excitatory projections were densest within and between nearby layers, including the principal feedforward pathway from L4 to L2/3, L2/3 to L5, and L5 to L6. In contrast, inhibitory projections between layers were sparse, consistent with the predominantly intralaminar organization of cortical inhibition. A weaker and broader feedback projection connected L6 back to L4, providing a recurrent pathway from deeper to more superficial cortex. These connectivity patterns follow the organization reported for mouse neocortex, in which excitatory projections form the principal interlaminar routes whereas most inhibitory axons remain within their local cortical layer [6,7,8,9,10,15,16].

The resulting network contained 96,760 synaptic connections distributed across 29 projection pathways. Synaptic weights followed the specified distributions, each weight entered the postsynaptic current through the dual-exponential kernel of Equation (2), and conduction delays increased with intersomatic distance (Figure 2f). We used the same wiring for all simulations, so we could not attribute differences in population responses across stimulus conditions to changes in network wiring.

### 3.3. L4 Stimulation Propagates Through the Cortical Hierarchy

We next examined whether input-layer stimulation engaged the recurrent network and how the response propagated across cortical layers. The composite drifting-grating and noise stimulus (Equation (9), with grating contrast set by Equation (8)) was delivered as an additive current to L4 pyramidal neurons, while activity was recorded throughout the network (Figure 3a). No other population received direct visual stimulation.

The stimulus produced a graded response across the cortical hierarchy (Figure 3c). At the highest stimulus gain, mean L4 pyramidal firing increased from 3.78 Hz during spontaneous activity to 15.35 Hz during stimulation, corresponding to a 306.5% increase. The response was progressively attenuated in downstream layers, with firing-rate increases of 52.3% in L2/3, 13.3% in L5, and 1.0% in L6. Activity generated by L4 stimulation therefore propagated through the feedforward pathway but became substantially weaker at successive stages, with L6 remaining close to its spontaneous firing rate.

The response magnitude depended on stimulus gain (Figure 3d,e). The three highest gains (10, 20 and 40 µA/cm^2^) increased L4 pyramidal firing by more than 10% relative to spontaneous activity, whereas the two lowest gains produced changes within the range of fluctuations generated by the background Ornstein–Uhlenbeck input (Equation (4)). Despite increased stimulus-driven activity, the network remained in an asynchronous-irregular regime across conditions. Population spectra were broadband and showed no dominant oscillatory peaks. We therefore characterized spectral responses using their aperiodic component rather than identifying or comparing individual oscillatory peaks [26,43]. Having established that L4 stimulation engaged the network and propagated beyond the input layer, we next asked whether population activity also depended on the spectral structure of the injected noise.

### 3.4. Spectral Slope Produces Graded Population Responses

We varied the spectral exponent (*β*) of the injected noise while holding the other stimulus properties constant (Figure 3b). We generated colored noise by filtering white noise in the three-dimensional Fourier domain with the radially symmetric power-law filter in Equation (6), allowing us to control the spectral exponent across two spatial and one temporal dimension. The filter exponent maps onto the conventional noise-color parameter via the naming identity in Equation (7), and the resulting stimulus spectra follow the power law in Equation (1). The five conditions spanned violet, blue, white, pink, and brown noise. We normalized each noise field to unit variance, so the conditions differed in the distribution of power across frequencies rather than overall noise amplitude.

Population responses changed systematically with *β*. Rather than producing five discrete response states corresponding to the five noise colors, the network showed graded changes along the spectral axis. For example, L2/3 interneuron firing increased by 0.041 Hz for each unit increase in *β* at gain 10 µA/cm^2^ (partial η^2^ = 0.19, *q* = 1.2 × 10^−7^, *n* = 45 simulations; Figure 3f). Similar relationships were observed across cortical layers and cell types; the strongest trends appeared in layer-4 pyramidal interval variability (η^2^ = 0.49, *q* = 3 × 10^−13^) and synchrony (η^2^ = 0.42, *q* = 6 × 10^−11^), and the layer-5 pyramidal spectral slope declined with *β* (η^2^ = 0.26, *q* = 2 × 10^−6^). After false-discovery-rate correction, 25 of 48 layer-by-cell-type comparisons showed significant linear relationships with *β* (*q* < 0.05). The color effect was carried predominantly by the interaction between color and cortical layer, which reshaped the laminar profile of population synchrony (partial η^2^ = 0.64, *q* = 1.5 × 10^−50^), temporal signal-to-noise (η^2^ = 0.61, *q* = 2.6 × 10^−46^), and firing rate (η^2^ = 0.61, *q* = 1.6 × 10^−46^), rather than shifting all layers uniformly. Color also interacted with noise contrast in the spectral measures (χ, η^2^ = 0.27; temporal SNR, η^2^ = 0.24; Figure 3g,h).

A linear model also adequately described the relationship between population responses and *β* over the tested range. Adding a quadratic *β* term did not improve any of the 80 models after correction for multiple comparisons. Within the tested range, the response varied approximately linearly with spectral slope, rather than showing evidence of preferential tuning to a particular noise color.

The magnitude of the standardized response differed from this continuous *β*-dependent trend. Standardized deviations from the grand mean ranked the five conditions as pink > brown > blue > white > violet (Figure 3i). Pink noise therefore produced the largest standardized response even though brown noise had the highest *β*. This difference arises because standardized response strength depends on both the displacement of the population response from the grand mean and its dispersion. Response variance differed across the five spectral conditions (Brown–Forsythe test, *F*_(4,175)_ = 14.02, *p* = 6 × 10^−7^). The two analyses therefore capture different aspects of the response. Regression against *β* measures the systematic change in the population mean along the continuous spectral axis, whereas standardized response strength measures how far each condition is from the grand mean relative to its response variability. A condition can therefore shift the population mean substantially while yielding a smaller standardized response if it also increases dispersion. However, a shift in the population mean alone does not establish that the stimulus condition can be identified from a population response. This would require multivariate decoding.

### 3.5. Spectral Slope Is Decodable from Population Activity

We asked whether the multivariate population response could identify the spectral condition. We classified the five noise conditions using a five-class nearest-centroid classifier based on 12 physiological features (Figure 4; see Section 2).

The population activity reliably decoded the spectral condition. We performed cross-validation at the simulation level, so we never used activity from the same simulation for both training and testing. A continuous decoder recovered *β*: the cross-validated correlation between predicted and true *β* was *r* = 0.83 (RMSE = 0.79 in *β* units), confirming a graded rather than categorical representation. Indeed, decoding performance varied across layers and cell types. L4 provided the strongest readout, followed by L2/3, whereas populations in L5 and L6 remained at or near chance (Figure 4a). Decoding accuracy for the L4 population was 69.6% (95% CI [55.2, 80.9]; *n* = 46). Because the five-way point estimate varies with fold assignment at this sample size, we report a confidence interval and use the continuous *β* decoder as the primary readout.

Interestingly, the five spectral conditions were not equally easy to distinguish (Figure 4b). Violet noise had the highest sensitivity (62%), whereas pink noise had the lowest (50%), with blue, white, and brown falling between these values. This pattern differed from the univariate response-strength measure in Figure 3i, where pink produced the largest standardized response and violet the smallest. Pink noise lies near the center of the *β* continuum and therefore has neighboring conditions on both sides, making it more difficult to distinguish as a discrete class despite its strong univariate response. Violet noise, by contrast, lies at one end of the continuum and was easier to identify despite its weaker univariate response. The magnitude of a univariate response and the separability of population states therefore capture different aspects of stimulus representation, as also observed in cortical responses to complex visual stimuli [48].

Decoding accuracy increased with noise contrast (Figure 4c). The higher the held-out level’s contrast, the more accurately the decoder labeled it, with accuracy rising step by step from the lowest noise contrast to the highest. Higher noise contrast therefore produced increasingly separable population representations of the spectral condition. This dependence on stimulus strength is consistent with a representation that is limited, at least in part, by the signal-to-noise ratio of the population response. It also relates to theoretical work showing that external noise can improve the detectability of structured signals in nonlinear systems [49].

### 3.6. Decoding Generalized to Noise Fields Not Used for Training

In our main design, each color was associated with a single noise field, which was presented at ten different contrast levels by multiplying that field by ten scaling factors. Thus, omitting one contrast level did not introduce a novel noise field: the decoder had already encountered the same field at the other nine contrast levels. Consequently, this manipulation tested whether a color remained recognizable when the contrast of a familiar noise field changed. It did not test recognition of a color in a previously unseen noise field, because no such field was included in the test set.

To test generalization across fields, we generated ten independent noise draws per color, each an independent white field filtered by the same power law, run at gain 10 µA/cm^2^ in both stimulation conditions across the full contrast series (100 simulations per draw). Transfer accuracy used leave-one-draw-out cross-validation, so no field appeared at any contrast in both training and test sets; the within-field comparison was recomputed on the same simulations with the same features. Color information transferred to fields the decoder had never been trained on, at a reduced level. L4 decoding was 30.5% under leave-one-draw-out (274/898; Wilson 95% CI [27.6, 33.6]; permutation *p* < 1 × 10^−4^; chance 20%), against 66.3% within field on the same simulations (Δ = −35.7%; bootstrap CI [−40.0, −31.5]; exact McNemar *p* < 1 × 10^−49^). Therefore, a substantial part of within-field decodability was specific to the noise field, while a smaller but reliable component generalized. The distance between transfer accuracy and chance reflects the component of the representation tied to spectral slope, whereas the distance between transfer and within-field accuracy reflects the component tied to the training field.

Two features of the transfer result indicate that the transferring component is spectral rather than incidental. Transfer accuracy rose with noise contrast, from 21% at the lowest level to 37% at the highest (Figure 4d), which a decoder that had learned particular fields would not show on unseen fields. Misclassifications concentrated on neighboring colors rather than scattering across the spectral axis, and a continuous decoder of *β* transferred at *r* = 0.43 (RMSE 1.31 *β* units). Both observations indicate that the decoder transfers position along the spectral continuum rather than color identity. Restricting the analysis to features shared with the main decoder gave a similar result (L4, 28.8%; *β* r = 0.46), so the finding does not depend on the feature set used.

### 3.7. The Spectral Representation Is Not Uniform Across Cortical Layers

We wondered whether the spectral structure was distributed uniformly across the cortical hierarchy. Because we delivered the stimulus only to L4, a uniform representation across layers would be consistent with passive propagation. In contrast, a stronger representation in a downstream layer would indicate that intracortical processing enhanced the input’s spectral structure.

The two measures we applied peak in different layers, and the difference is informative. In the univariate analysis, *β* explained the largest share of response variance in L2/3 (partial η^2^ = 0.54, gain 40 µA/cm^2^), after which the effect size declined progressively along the feedforward cascade from L2/3 to L5 and L6, by an average of 0.070 η^2^ per layer (Figure 5a). By this measure, *β* had its strongest effect on individual response metrics one synaptic stage beyond the layer receiving the external input.

Decoding ordered the layers differently. Accuracy was highest in L4, second in L2/3, and fell in the deeper layers (Figure 5b). The same order appeared in both decoding analyses, which differ only in how populations are grouped: Figure 5b pools cell types within a layer, whereas Figure 4a treats each layer × cell-type × gain combination on its own. The two measures captured different aspects of the response. Partial η^2^ measures how much variance in a response metric is associated with *β*, whereas decoding measures how separable the five conditions are in the full 12-feature population space. L4 showed the highest decoding accuracy, indicating the most separable population states, whereas L2/3 showed the largest proportional effects of *β* on individual metrics. Thus, the two measures point to a similar conclusion: spectral information is distributed unevenly across layers and does not simply decrease with distance from the input.

The laminar profile therefore does not resemble a simple attenuation of the input as it propagates through the network. In the univariate measure, the spectral signature became more prominent from L4 to L2/3 before declining in deeper layers, and in both measures deep layers fell well below the two superficial ones. The simulations did not identify which specific recurrent connections or cellular mechanisms generated this profile, so we should interpret the result as a property of the network architecture and dynamics rather than evidence for a particular mechanism.

### 3.8. Layer-4 Inhibition Does Not Reduce Spectral Discriminability

We next asked whether feedforward inhibition in L4 altered spectral structure representation. In L4, external sensory input recruits both excitatory and fast-spiking inhibitory neurons, so we compared two otherwise identical stimulation conditions that differed only in whether the external drive was also delivered to L4 interneurons. Network connectivity, stimuli, and background activity were identical between conditions.

Recruiting L4 inhibition did not detectably change the relationship between mean firing rate and spectral slope. In a mixed-effects model with run-level random slopes, the condition × spectral-slope interaction was 0.016 (95% CI [−0.24, 0.21], *p* = 0.89). The main effect of spectral slope on firing rate was also not distinguishable from zero (0.094, 95% CI [−0.06, 0.25]). Therefore, mean firing rate provided no evidence that L4 inhibition systematically altered responses to different noise colors (Figure 5c).

Decoding accuracy was likewise unchanged. Recruiting L4 inhibition did not significantly change five-way decoding accuracy, and the confidence intervals for the two stimulation conditions overlapped (Figure 5d). Recruiting feedforward inhibition therefore neither improved nor degraded the separability of population states, under either the univariate or multivariate measure. Inhibition can regulate cortical gain and reduce internally generated variability, either of which could affect the geometry of population responses without producing a corresponding change in their mean response [50,51,52,53]. However, because we switched inhibition on as a block rather than varying it systematically, the present simulations cannot distinguish between these possibilities. More generally, reduced correlations do not necessarily imply greater information: decorrelation improves a readout only when the removed correlations limit stimulus discriminability [54,55]. We conclude that recruiting L4 inhibition left the five spectral conditions as separable as they were without it.

### 3.9. Spectral Representations Are Stable Across Stimulus Gain

We quantified whether the relative representation of the five spectral conditions was preserved across stimulus strength. We repeated the same experimental design across five stimulus gains spanning a 16-fold range (2.5–40 µA/cm^2^), and we calculated the strength of each spectral condition independently at each gain (Figure 5e,f).

At gains that produced robust network responses, the relative ordering of the spectral conditions was generally preserved. Agreement with the pooled ordering reached Kendall’s τ = 0.80 at the highest gain (40 µA/cm^2^) and τ = 0.60 at the second-highest gain (20 µA/cm^2^). This indicates that increasing stimulus drive changed the magnitude of the population response without fully reorganizing the relative representation of the different spectral conditions.

Stability was weakest at the lowest gain that produced a measurable response. At 10 µA/cm^2^, the ordering of the five colors differed substantially from that observed at higher gains (τ = 0.59), and the overall network response was considerably weaker. Because Kendall’s τ is based on only ten pairwise comparisons among five conditions, small changes in the relative magnitude of neighboring color responses can substantially alter the coefficient. This instability therefore reflects the difficulty of reliably ordering the colors by the magnitude of their univariate responses, rather than a loss of separable population-level information. Indeed, decoding, which uses all 12 features simultaneously and is evaluated by cross-validation, remained well above chance and above the permutation null at this gain. Thus, although the univariate response ranking was unstable, spectral information remained detectable in the population response.

The two lowest gains did not produce reliable increases in activity above spontaneous background and did not show stable spectral rankings. These conditions provide a useful lower-bound control: a consistent ordering of the spectral conditions emerged only when the stimulus produced a measurable cortical response. The spectral representation was stable across the stronger stimulus gains but became less reliable as the network approached its effective response threshold.

### 3.10. The L4 Spectral Signal Is Feedforward, and Recurrence Shapes Its Laminar Spread

To test whether the decodable color signature required recurrent processing, we compared the intact network with two ablations run in the same simulator, with identical neuron models, tonic currents, background activity, stimuli, L4 delivery, features, and decoder; only the synaptic weight matrices changed. In the disconnected network all synaptic weights and the interneuron gap junctions were set to zero. In the no-intralaminar network, the sixteen within-layer pathways and the gap junctions were set to zero, leaving the interlaminar projections intact (Figure 6).

L4 color decoding was essentially unchanged by either ablation: 69.6% in the intact network, 63.0% when the network was disconnected (Δ = 6.5%; exact McNemar *p* = 0.45; *n* = 46 matched pairs), and 63.0% without intralaminar recurrence (Δ = 6.5%; *p* = 0.51). The spectral signal available at L4 was therefore largely feedforward, carried by the stimulus and by the intrinsic dynamics of L4 cells rather than constructed by recurrent interaction. This is expected on information-theoretic grounds: the spectral slope was imposed on the input to L4, so recurrence cannot add information about *β* that its input does not carry.

Recurrence changed how that signal was distributed across layers. Disconnecting the network dropped L2/3, L5, and L6 to chance (~22%; chance 20%), since without synapses these layers receive no input. Removing only intralaminar recurrence, while leaving the interlaminar pathways intact, instead raised deep-layer decoding above the intact network: L6 from 39.1% to 78.3% (Δ = 39.1%; *p* < 0.001) and L5 from 45.7% to 71.7% (Δ = 26.1%; *p* = 0.012). Local recurrent competition within a layer therefore limited how much of the feedforward signal reached the readout. We verified readout sensitivity for each ablation. Both ablated networks remained active, with median firing rates of 4.8 Hz in the disconnected network and 10.4 Hz without intralaminar recurrence; no cells were silent. We also verified that the decoder could read information from these data. When trained and tested on the same simulations, it reached 73.9%, showing that the features were separable and that the classifier could use them. The disconnected network also decoded noise contrast at 13.0% in the deep layers, above the 10.0% chance level, while color decoding was at chance. Thus, the chance-level color results in the other layers reflect the absence of a color signal rather than a failure of the decoder. These two control simulations addressed different levels of processing. The disconnected network asked whether synaptic interactions were required to express the spectral signal at L4, whereas the feedforward model asked whether the stimulus could be decoded without the intrinsic dynamics of the L4 cell models. Together, they indicate that spectral information at L4 depended on cellular dynamics rather than on recurrent synaptic interactions or the amplitude cue alone.

## 4. Discussion

The spectral structure of external noise remained detectable after propagation through a biologically inspired recurrent cortical network. Population responses changed systematically with the spectral slope of the input, and the five spectral conditions could be classified from multivariate activity. The representation was unevenly distributed across layers, with decoding highest in the stimulated L4 and the largest univariate effect of *β* one stage downstream in L2/3, became more separable with increasing noise contrast, and remained relatively stable across the stronger stimulus gains tested. Together, these results show that recurrent cortical dynamics did not simply remove the input’s statistical structure. Instead, they transformed it into population activity from which that structure remained recoverable. This result should not be interpreted as evidence that the network explicitly estimated the spectral exponent *β*. The network was not trained to recover *β*, no neuron was specifically tuned to it, and no model component received an objective related to the input spectrum. Instead, changes in *β* were reflected as coordinated changes across several network activity properties, including firing rate, membrane potential variance, spectral power, and the aperiodic component of the population spectrum. We applied the decoder after the simulations, so it tested whether the population response contained information about the spectral condition, rather than modeling how a biological circuit would read out that information. Successful decoding shows that the information was available in the network activity. It does not establish that downstream circuits extract or use it during perception or behavior.

A similar distinction is important for the term “preservation.” The spectral signature was not transmitted unchanged from L4 to the other cortical layers. Synaptic filtering, nonlinear membrane dynamics, and recurrent excitation and inhibition continuously transformed neural activity. Activity in L2/3 was therefore not a copy of the activity entering L4. What persisted was information about the input’s statistical properties. In this sense, the network preserved the spectral structure at the level of population statistics rather than at the level of individual neural spikes. This distinction is consistent with the general view that cortical processing is transformational rather than purely transmissive [56]. It also provides the main rationale for this approach: although recurrent processing can make individual spikes hard to track, statistical properties of population activity can still reveal the structure of the original input. Whether this form of information preservation occurs in biological cortex remains to be tested.

The different responses to the five spectral conditions suggest that this representation arises from the network’s temporal dynamics rather than from a categorical encoding of noise color. The network responded strongest to pink noise, followed by brown, blue, white, and violet noise. Pink and brown noise contain relatively more power at low frequencies, which may interact more strongly with the temporal filtering imposed by synaptic and cellular dynamics. The delivered temporal spectra make this interpretation concrete. The strongest responses occurred for inputs with greater low-frequency structure, indicating that the response related to the stimulus’s temporal correlations rather than simply its nominal color. Synaptic time constants, dendritic integration, calcium-dependent conductances, and recurrent interactions all constrain the temporal frequencies to which the network can respond. Low-frequency-rich input can therefore produce larger changes in firing rate, membrane-potential variance, and population spectral power, whereas these intrinsic dynamics may attenuate faster components. This interpretation is consistent with the network behaving as a dynamical system whose frequency-dependent response is determined by its cellular and synaptic time constants. Changing *β* alters the distribution of stimulus power across frequencies, thereby changing how much of that power falls within the frequency range to which the network is most responsive. The resulting differences in population activity can then appear simultaneously in several response measures [41,43,57]. However, the present simulations do not establish that temporal filtering is the mechanism responsible for the observed ordering. In future simulations, we could test this directly within the same framework by systematically varying synaptic or dendritic time constants and asking whether the relative representation of the five spectral conditions changes accordingly.

The generalization test defines an important limit on the decoding result. Decoding generalized to noise fields not used for training, but with much lower accuracy than within-field decoding. This difference was expected because, in the main design, each color was represented by a single field and all contrast levels were scaled versions of that field. A decoder trained across contrast levels could therefore use both spectral information and field-specific features. The transfer result shows that part of the population response reflected spectral slope rather than the particular noise image. Transfer accuracy increased with noise contrast, and errors were concentrated between neighboring colors, consistent with a graded representation of *β*. An appropriate conclusion is therefore narrower than simply stating that spectral slope was decodable: population activity contained information about *β* that generalized across noise fields, but it also contained field-specific information. Using multiple independent fields per condition is necessary to separate these two sources of decoding.

The ablations showed that the L4 signal did not require recurrent synaptic interaction. Removing all synapses did not change L4 decoding, indicating that the input-layer spectral information was already in the stimulus and could be expressed by L4 neurons’ intrinsic dynamics. That expression is not automatic. A feedforward model receiving the same stimulus through generic leaky integrate-and-fire units did not support decoding, showing that membrane filtering and spike threshold alone were insufficient. The two controls therefore separate the stimulus contribution from cellular dynamics and recurrent interactions. The stimulus became decodable when L4 cell models processed it, whereas adding recurrence did not substantially alter its representation at L4. Recurrence therefore did not generate the information about *β*. It determined how that information was distributed across downstream layers. The disconnected network produced chance-level decoding in L2/3, L5, and L6 because these layers received no input. In contrast, removing only intralaminar connections while preserving the interlaminar pathways increased decoding in L5 and L6. Thus, local recurrent interactions influence how much of the feedforward spectral signal is accessible at each stage. The laminar profile is therefore not a simple attenuation of the input signal. It reflects the processing that occurs within each layer. Whether the biological cortex shows a similar organization remains to be tested.

The aperiodic component of the population spectrum requires a similar caution. Aperiodic spectral slope often serves as a proxy for excitation–inhibition balance [41], but recent modeling work shows this relationship is not general. Changes in inhibition can steepen or flatten the population spectrum depending on the operating regime, with no reliable mapping between spectral slope and the excitatory-to-inhibitory conductance ratio across the regimes examined [58]. We therefore treat the aperiodic exponent here as a descriptor of population activity and as a feature available to the decoder, rather than a measure of excitation–inhibition balance. This distinction matters because the present simulations show that the population spectrum changes with the input’s spectral structure, but they do not establish a specific biophysical mechanism underlying those changes.

The relation between these findings and efficient coding is also suggestive but should not be interpreted as evidence for an ‘efficient-coding mechanism’. Natural visual inputs contain substantial scale-free structure, and efficient coding theories propose that sensory systems adapt to regularities in their inputs while reducing redundancy [59,60,61]. Natural scenes exhibit approximately 1/f-like statistics across spatial and temporal scales [62,63], and several experimental studies have shown that early visual circuits are sensitive to these regularities. For example, geniculate neurons preferentially whiten the temporal statistics of natural movies while leaving responses to white-noise stimuli relatively unchanged [64], coding in the fly retina matches the contrast distribution of the natural environment [65], and response properties in mouse V1 have been interpreted as consistent with efficient coding of natural images [33]. Our model, however, does not implement efficient coding. The network was not exposed to natural images, did not learn connectivity from sensory experience, and had synaptic plasticity disabled. Therefore, it had no explicit prior or optimization process that favored natural visual statistics. The relevant observation is narrower: a network constructed from cortical cellular and circuit mechanisms exhibited stronger population responses to input with an approximately 1/f-like temporal structure, even in the absence of learning. This suggests that some biases often associated with sensory coding could arise from the circuit’s intrinsic dynamics, without requiring those statistics to be learned. Whether experience or plasticity would strengthen, weaken, or otherwise reshape this bias remains an open question.

The power-law structure imposed on the stimulus should also be distinguished from power-law structure that can emerge intrinsically in population activity. Mouse V1 populations exhibit a power-law relationship in the variance of their principal components, and this structure persists even when stimulus statistics are whitened [66]. Our result concerns a different relationship: a controlled spectral property of the input is transformed into population activity in the recurrent network. The similarity in scaling properties therefore does not imply that the network-generated population structure is simply inherited from the stimulus. Rather, it highlights that stimulus statistics and intrinsic population dynamics can both contribute to the statistical structure observed in neural activity.

The decoding results further indicate that the spectral representation is distributed across the population rather than concentrated in a single response measure. Neurons operating in the high-conductance regime receive continuous synaptic bombardment and exhibit substantial trial-to-trial variability [22,25]. Such variability can obscure the relationship between an individual neuron’s activity and a particular stimulus statistic. At the population level, however, different response dimensions can be combined so that stimulus-related structure becomes more readily recoverable. This is consistent with the broader concept of suprathreshold stochastic resonance, in which reliable representations can emerge from populations of individually variable elements [49,67].

The feature analysis supports a similar interpretation. No single physiological variable accounted for the full spectral representation: the best individual feature decoded substantially below the full population vector, whereas removing any one feature produced only a small reduction in performance. The representation was therefore distributed across multiple measures, including firing rate, response variability, membrane dynamics, and spectral properties. These results indicate that the population state, rather than any single physiological variable, carried spectral information. The combination of weak single-feature decoding and a small leave-one-out penalty is consistent with a redundant population representation in which multiple physiological features contribute to the same stimulus information.

L4 inhibition further shows why the spectral representation cannot be understood from mean firing rates alone. Recruiting fast-spiking interneurons changed neither the dependence of mean firing rate on spectral slope nor decoding accuracy, and the confidence intervals for the two stimulation conditions overlapped. One possible explanation is that inhibition altered the relationships among neurons rather than simply suppressing their responses. Shared excitation accompanied by delayed inhibition can reduce correlations between neurons [68], consistent with theoretical and experimental evidence that inhibition can decorrelate cortical activity [26,69,70]. Reduced correlation, however, does not necessarily increase information. Decorrelation improves the information available to a readout only when the correlations being removed interfere with stimulus discrimination; correlations that carry stimulus-related structure may instead contribute to the representation [54,55]. The present result is therefore best stated conservatively: recruiting L4 inhibition did not compromise the separability of the spectral conditions, despite its limited effect on the mean response. An alternative explanation is that inhibition reduced internally generated variability rather than primarily decorrelating the population. Feedforward inhibition regulates cortical gain, sharpens temporal responses, and limits excessive excitation [51]. Inhibitory stabilization can also suppress internally generated fluctuations while preserving stimulus-driven responses [50,52,53]. Either effect could make stimulus-related structure easier to recover without increasing the mean response. Because inhibition was manipulated as an on/off condition rather than ‘titrated’ across conductance levels, the present simulations cannot distinguish between these mechanisms.

The network’s asynchronous, irregular operating regime may also contribute to the representation’s robustness. Low pairwise synchrony limits internally generated population correlations and may make externally driven statistical structure easier to recover [26]. Conversely, in more strongly coupled networks, internally generated slow fluctuations could occupy population activity dimensions that overlap with those driven by the external input, reducing the recoverability of the external input’s spectral structure [71]. This suggests a direction for future simulations: varying inhibitory conductance and network coupling while measuring both pairwise correlations and decoding accuracy could determine whether changes in spectral discriminability are driven primarily by decorrelation, suppression of internal variability, or shifts in the overall operating regime.

The representation was also not distributed uniformly across the cortical hierarchy. The stimulus entered the network at L4, which gave the most separable population states, while the largest univariate effect of *β* on individual response metrics appeared one stage downstream in L2/3; both measures then declined through the deeper layers. This pattern argues against a simple feedforward relay. L4 receives the ascending sensory drive, whereas recurrent interactions in superficial cortex integrate this input through local and horizontal connections before information is passed to other cortical stages [56]. In the present network, these interactions produced a population state in which the statistical structure of the input was particularly easy to recover. The simulations do not identify which specific connections or cellular mechanisms generated this laminar profile, and whether a comparable laminar gradient exists in biological cortex remains an open question.

The increase in decoding accuracy with noise contrast connects these findings to the broader literature on stochastic resonance, although the computational question here differs. In nonlinear systems operating near threshold, external noise can improve transmission of a weak signal by increasing the probability of threshold crossing [35,72,73,74]. The effect can also depend on the noise’s temporal structure, with low-frequency-rich noise producing stronger effects than white noise in some systems [75,76]. Here, however, the noise was itself the stimulus property of interest. We did not ask whether noise improved the detection of another signal; we asked whether the statistical structure of the noise became represented in recurrent population activity. It did, and this representation became more recoverable as noise contrast increased. This distinction matters for how experiments interpret externally applied noise. Researchers often treat noise as a source of variability that limits sensory coding [77]. At the same time, neural variability can reflect structured network dynamics and contribute to computation [57,67]. Our simulations support the latter possibility in this network: recurrent processing did not simply discard externally imposed variability. Its statistical structure remained embedded in population activity, and its representation became increasingly recoverable as the input magnitude increased.

Several limitations constrain our conclusions. The network was biologically inspired but remains a simplified cortical circuit. We did not include neuromodulation, long-range corticocortical interactions, neuronal diversity, or experience-dependent plasticity, which could alter the representation of stimulus statistics. All analyzed simulations ran on one fixed network realization, so the results do not quantify variability across independently generated wiring; reported statistics treat the stimulus draw within this fixed network as the inferential unit. The effective L4 input amplitude also rose monotonically with *β*. This spread arises from sampling an equal-variance field through a finite interpolation aperture rather than from the stimulus construction itself. Because the feedforward model preserved this amplitude difference but lacked both cellular and network dynamics, the amplitude cue alone cannot account for color decoding. The exponent *β* should likewise be interpreted as an ordered parameter describing the spatiotemporal stimulus, not as the temporal slope of the input reaching an individual L4 neuron. Neuronal surface density also fell by a factor of 4.8 from L2/3 to L6 because each shell carried the same number of cells at a different radius; therefore, laminar differences cannot be attributed to synaptic organization alone. We also kept the stimulus manipulation deliberately simple: we added synthetic colored noise linearly to a drifting grating. This provided precise control over spectral slopes but captured only one statistical dimension of natural visual input. Within the main design, each color was represented by a single noise field, so a large part of the within-field decoding accuracy was specific to that field; the separate transfer experiment quantifies how much survives a change in field, and it is the transfer number, not the within-field number, that bears on spectral slope as such. Inhibition was switched on or off rather than varied in graded steps, so we cannot separate a change in decorrelation from a change in internally generated variability. We also defined the imposed stimulus spectrum only below 30 Hz, whereas we fitted the aperiodic exponent over 1–100 Hz, so most of the fitted range reflects network activity rather than the stimulus. We fitted the aperiodic exponent by ordinary least squares over linearly spaced frequency bins, which weighs the high-frequency decade most heavily; the procedure was identical across conditions, so this affects absolute exponents rather than between-condition comparisons. We also imposed cellular heterogeneity functionally rather than mechanistically: cells differed in their tonic depolarization, and therefore in their operating point, but not in their underlying conductances. Finally, decoding indicates that the population response contains information about the spectral condition but does not show that a downstream biological circuit extracts or uses it during perception or behavior.

These limitations suggest several direct extensions of the present model. Manipulating synaptic and dendritic time constants could test whether the circuit’s temporal filtering properties determine the ordering of spectral responses. Varying inhibitory conductance while measuring both correlations and decoding could distinguish decorrelation from suppression of internally generated variability. Repeating the simulations across independently generated networks would establish how robust the observed effects are to network architecture, while systematically changing the L4 → L2/3 connectivity could test whether the superficial peak depends on connection density. Equalizing neuronal surface density across shells would separate laminar packing from laminar connectivity as sources of the observed profile. Enabling plasticity would further test whether experience strengthens the apparent bias toward 1/f-like input statistics. At the experimental level, recordings from the visual cortex could test whether biological populations show comparable spectral representations and whether their laminar organization changes with behavioral state or stimulus statistics. The same framework could also be extended beyond spectral slope to other defined properties of sensory input, including temporal autocorrelation, higher-order moments, and spatial correlations.

## 5. Conclusions

Our network simulations showed that recurrent cortical processing can still detect information about the statistical structure of external variability. Network dynamics transformed the input and distributed this information unevenly across cortical layers, with spectral discriminability increasing with noise contrast. The ablation results showed that the spectral signal was already present at the input layer, while recurrent interactions shaped how much of this information was accessible downstream.

The main result is therefore not that the network preserved the stimulus spectrum itself. Rather, population activity retained information about its spectral slope despite multiple stages of recurrent processing. This provides a practical framework for studying information propagation in recurrent circuits: instead of tracking individual spikes across successive transformations, one can define and use a statistical property of the input to ask how that property is expressed and transformed in the population activity.

## Figures and Tables

**Figure 1:**
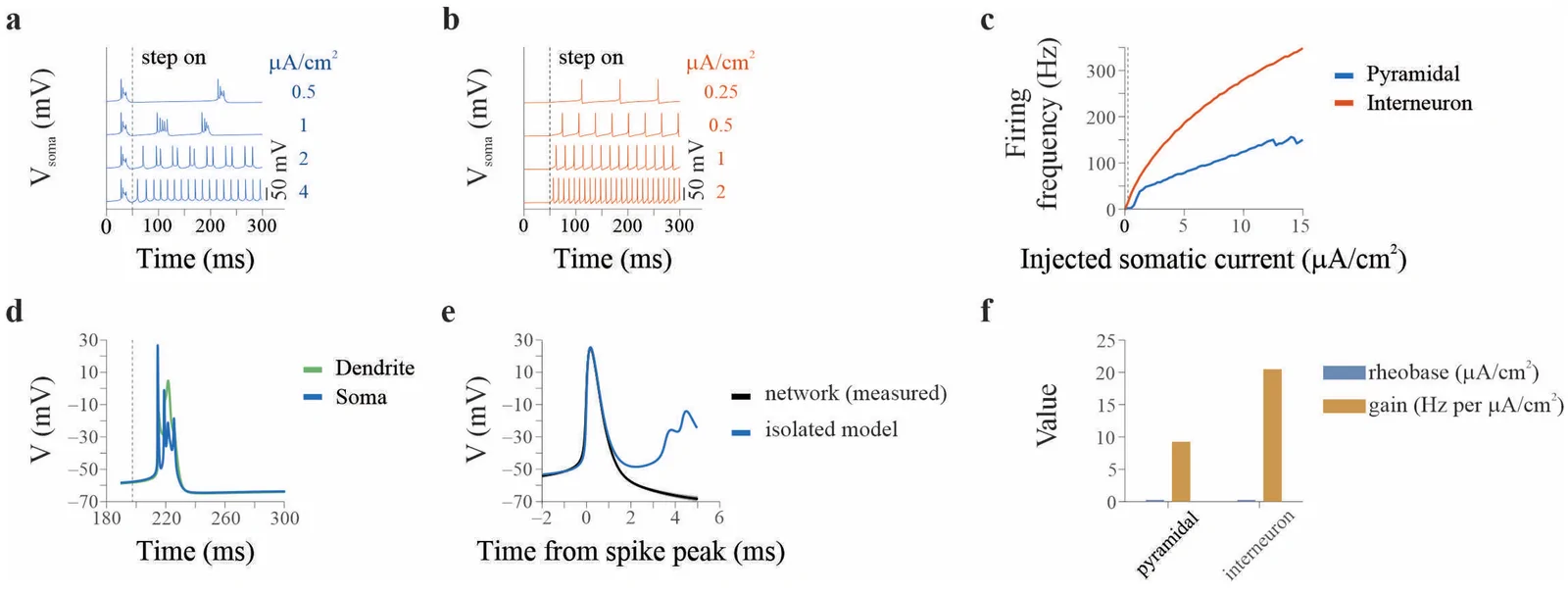
Intrinsic properties of the two neuron models. (**a**) Somatic membrane potential of an isolated pyramidal neuron (Pinsky–Rinzel model) during 600 ms current injections (0–15 µA/cm^2^; 0.25 µA/cm^2^ increments). (**b**) Somatic membrane potential of an isolated fast-spiking interneuron (Wang–Buzsáki model) under the same stimulation protocol. (**c**) Frequency–current (f–I) relationships for both neuronal models. (**d**) Somatic and dendritic membrane potentials during a representative pyramidal-cell burst, illustrating dendritic calcium-dependent bursting dynamics. (**e**) Mean spike waveform of pyramidal neurons recorded in isolation (black) and during network activity (gray; mean ± SD, *n* = 186 spikes). (**f**) Summary of rheobase and firing-rate gain for both neuronal models. Neuronal dynamics follow the formulations of Pinsky and Rinzel [2] and Wang and Buzsáki [3].

**Figure 2:**
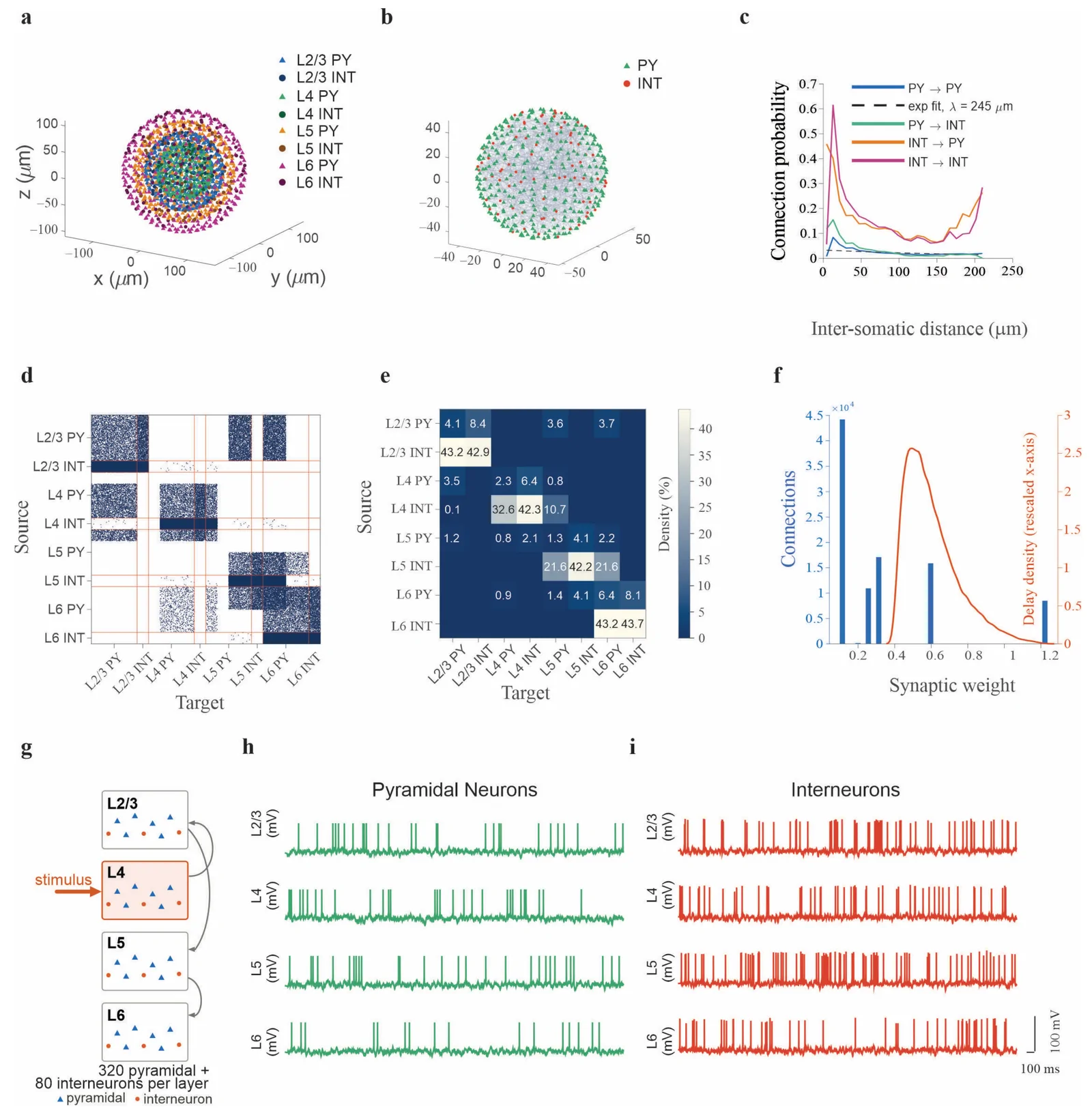
Architecture of the recurrent cortical network. (**a**) Spatial arrangement of the 1600 neurons across four concentric spherical shells representing cortical layers. We positioned neurons using a spherical Fibonacci lattice. Triangles indicate pyramidal neurons (320 per layer), and circles indicate fast-spiking interneurons (80 per layer). (**b**) Representative within-layer connectivity in L4, showing 800 randomly selected synaptic connections. (**c**) Connection probability as a function of intersomatic distance for the four within-layer connection classes (PY → PY, PY → INT, INT → PY, and INT → INT). Dashed line, exponential fit (λ = 261 µm). (**d**) Adjacency matrix of the complete network (1600 × 1600), with neurons ordered by cortical layer and cell type. (**e**) Connection-density matrix for all source–target layer and cell-type pairs (rows, presynaptic populations; columns, postsynaptic populations). (**f**) Distribution of synaptic weights and axonal conduction delays across the 96,760 synaptic connections. All analyzed simulations used this single network realization. (**g**) Laminar architecture and stimulus entry. The composite drifting-grating-plus-noise stimulus (Equation (9)) was injected into L4 pyramidal neurons; other layers received it indirectly via the connections in (**d**,**e**). Arrows indicate feedforward (L4 → L2/3 → L5 → L6) and feedback (L6 → L4) pathways. (**h**) Somatic membrane potential of one representative pyramidal neuron per layer during a single simulation. Traces are vertically offset. All show the same 1-s window. (**i**) As in (**h**), for one representative fast-spiking interneuron per layer. Same simulation, window, and scale bars.

**Figure 3:**
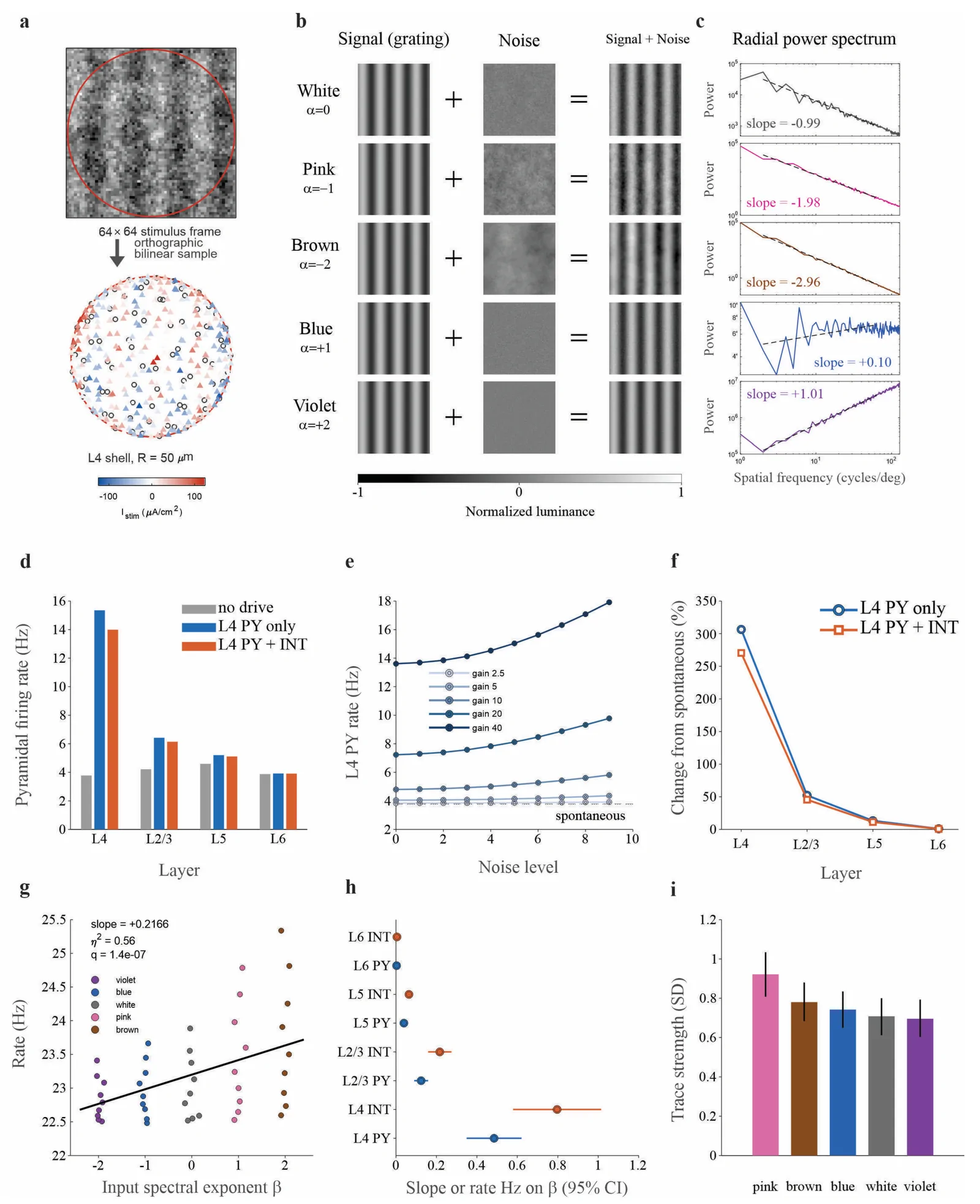
Layer-4 stimulation propagates through the network and preserves the input’s spectral structure. (**a**) Schematic of stimulus delivery. We projected a drifting grating, added linearly to colored noise, onto the L4 neuronal population by mapping each neuron to the stimulus image and converting pixel intensity into an additive input current. In this configuration, only L4 pyramidal neurons received direct stimulation. (**b**) Example stimuli for the five noise colors (violet, blue, white, pink, and brown). Columns show the drifting grating, the colored-noise field, the resulting composite stimulus, and the corresponding radially averaged power spectrum. (**c**) Mean pyramidal firing rate across cortical layers under spontaneous conditions and during L4 stimulation (gain = 10 µA/cm^2^). Values are averaged across all noise colors and noise levels. (**d**) L4 pyramidal firing rate as a function of stimulus gain. Dashed line indicates spontaneous activity. (**e**) Percentage increase in firing rate relative to spontaneous activity across cortical layers for the two stimulation conditions. (**f**) Example relationship between L2/3 interneuron firing rate and the input spectral exponent (*β*) at the highest stimulus gain. Line, least-squares fit; shaded area, 95% confidence interval. (**g**) Regression slopes (±95% confidence intervals) relating response magnitude to *β* for all layer and cell-type combinations. (**h**) Slope of firing rate on *β* for every combination of cortical layer and cell type, shown as a laminar map. (**i**) Strength of the spectral signature for each noise color, quantified as the standardized deviation from the grand mean (bootstrap 95% confidence intervals).

**Figure 4:**
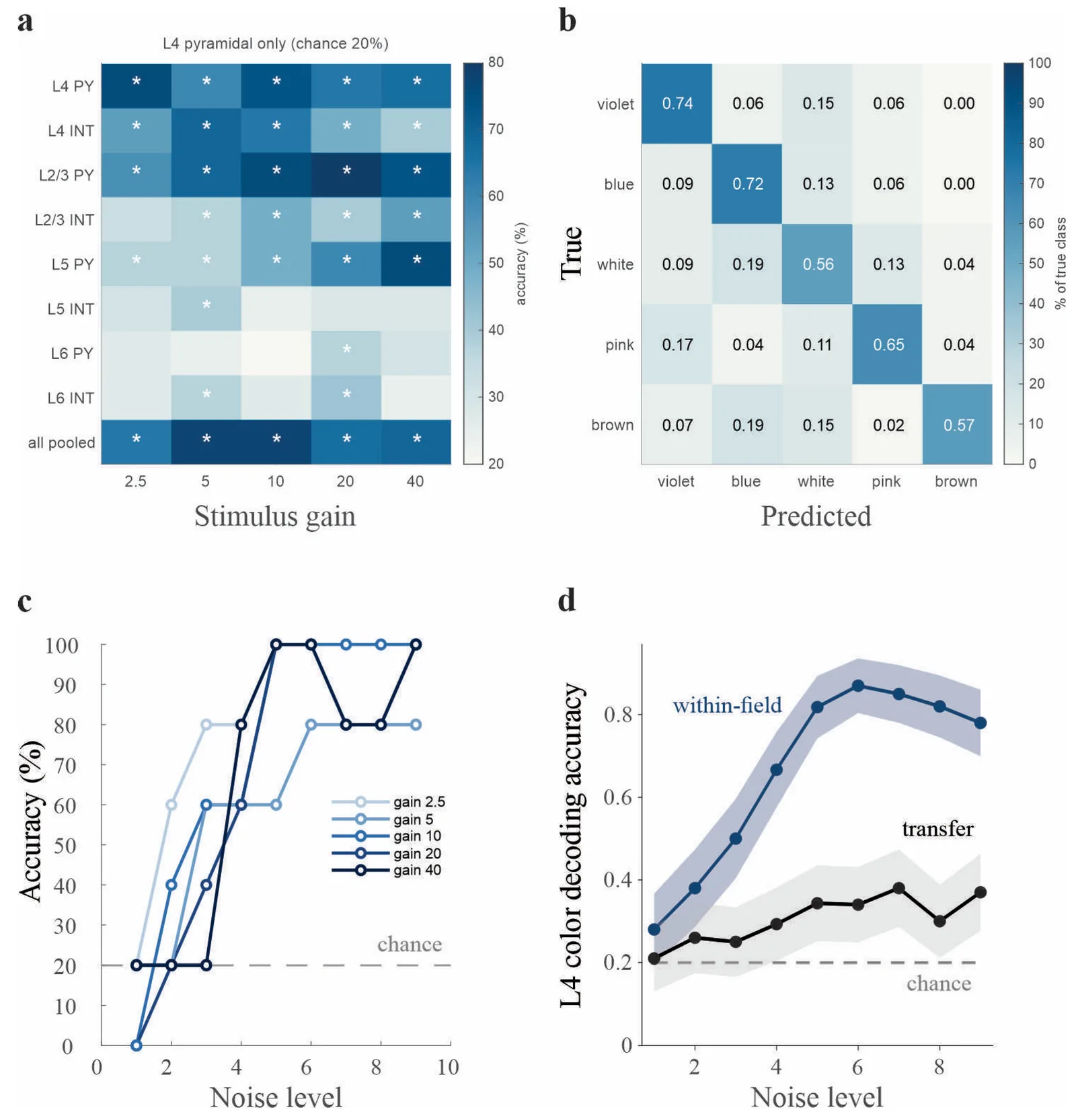
Population activity encodes the spectral structure of the input. Five-way nearest-centroid classifier trained on a 12-feature population response vector averaged over four 1-s analysis windows per simulation. Chance performance = 20%. Cross-validation followed a leave-one-noise-level-out scheme, training on the remaining non-zero noise levels and testing on the held-out level. (**a**) Classification accuracy for each cortical layer, cell type, and stimulus gain under L4 pyramidal-only stimulation. Asterisks mark decoders exceeding a label-permutation null (2000 permutations) after false-discovery-rate correction (*q* < 0.05). (**b**) Confusion matrix pooled across the 6 population(s) that exceeded the permutation null after false-discovery-rate correction at gain 10. Rows give the true stimulus color, columns the predicted color; diagonal entries are per-color sensitivity (violet 0.74; blue 0.72; white 0.56; pink 0.65; brown 0.57). (**c**) Classification accuracy of the L4 PY against the held-out noise level, one line per stimulus gain (2.5, 5, 10, 20, 40 μA/cm^2^). Dashed line, chance. *n* = 45 simulations per decoder at gain 10. (**d**) L4 decoding accuracy as a function of noise contrast under two cross-validation schemes. Within-field: training and testing use the same noise field at different contrasts, as in (**c**). Transfer: training uses nine independent noise fields, and testing uses a tenth that the decoder never saw. The transfer curve remains above chance at every contrast and rises with it, indicating a representation of spectral slope rather than the training fields; the gap between the curves reflects the within-field performance specific to each field. Shaded bands are Wilson 95% confidence intervals.

**Figure 5:**
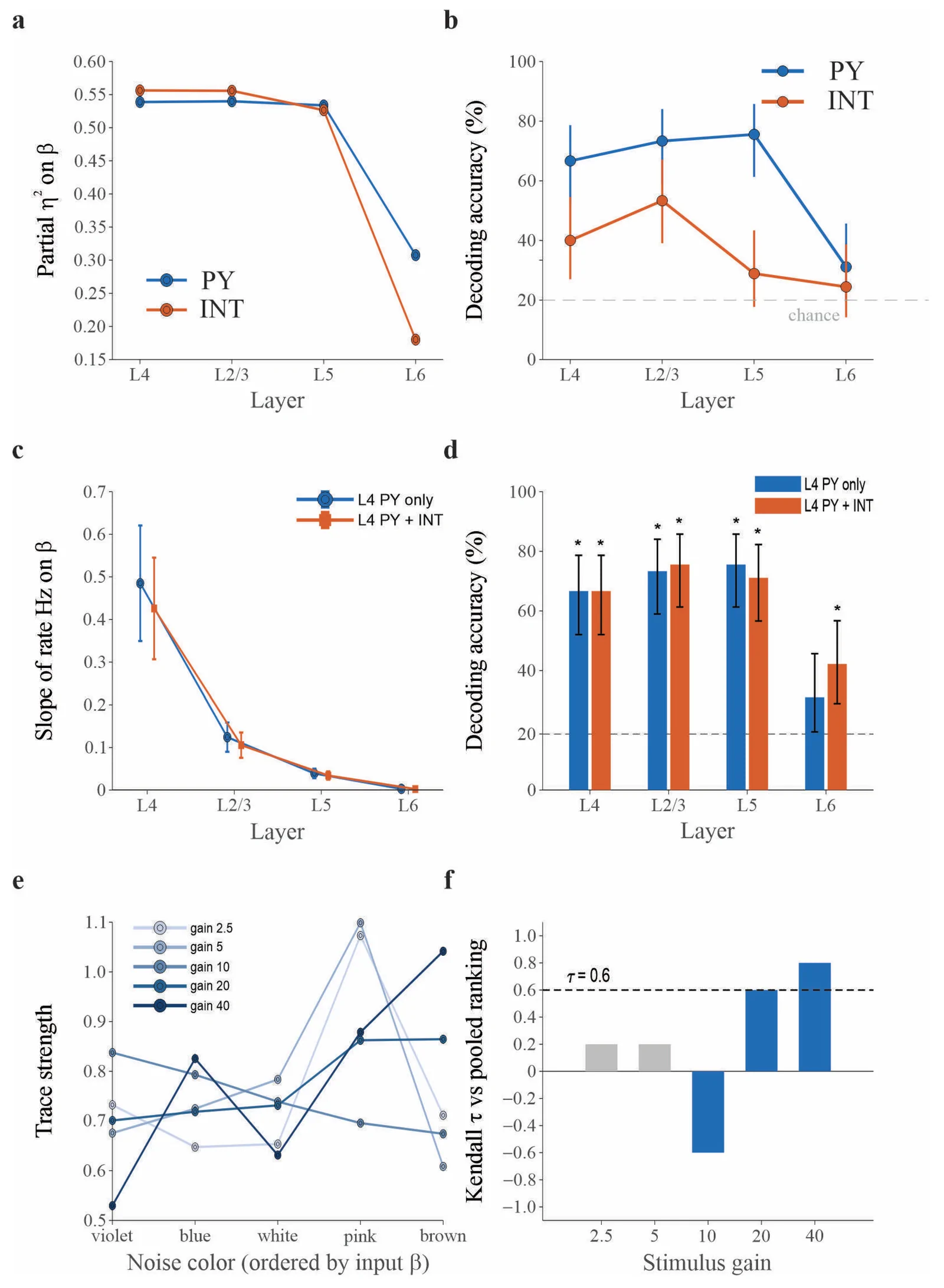
Laminar distribution, modulation by feedforward inhibition, and robustness of the spectral signature. Cortical layers are ordered along the feedforward cascade (L4 → L2/3 → L5 → L6). (**a**) Effect size (partial η^2^) of the relationship between firing rate and the input spectral exponent (*β*) for pyramidal neurons and interneurons at the highest stimulus gain (40 µA/cm^2^). (**b**) Accuracy across cortical layers at gain 40 μA/cm^2^ under L4 pyramidal only stimulation, plotted separately for PY and INT populations. (**c**) Regression slopes (±95% confidence intervals) relating firing rate to *β* under the two stimulation configurations: L4 pyramidal neurons alone and L4 pyramidal neurons plus interneurons. (**d**) Accuracy across cortical layers for PY populations at gain 40 μA/cm^2^ under the two L4 stimulation configurations (L4 pyramidal only vs. L4 pyramidal plus interneurons). Asterisks indicate statistically significant differences (*p* < 0.05). (**e**) Spectral-signature strength for each noise color, computed independently at each stimulus gain. (**f**) Stability of the color ordering across stimulus gains, quantified by Kendall’s τ-b relative to the pooled ordering. Filled bars indicate gains producing reliable network responses; open bars indicate subthreshold gains.

**Figure 6:**
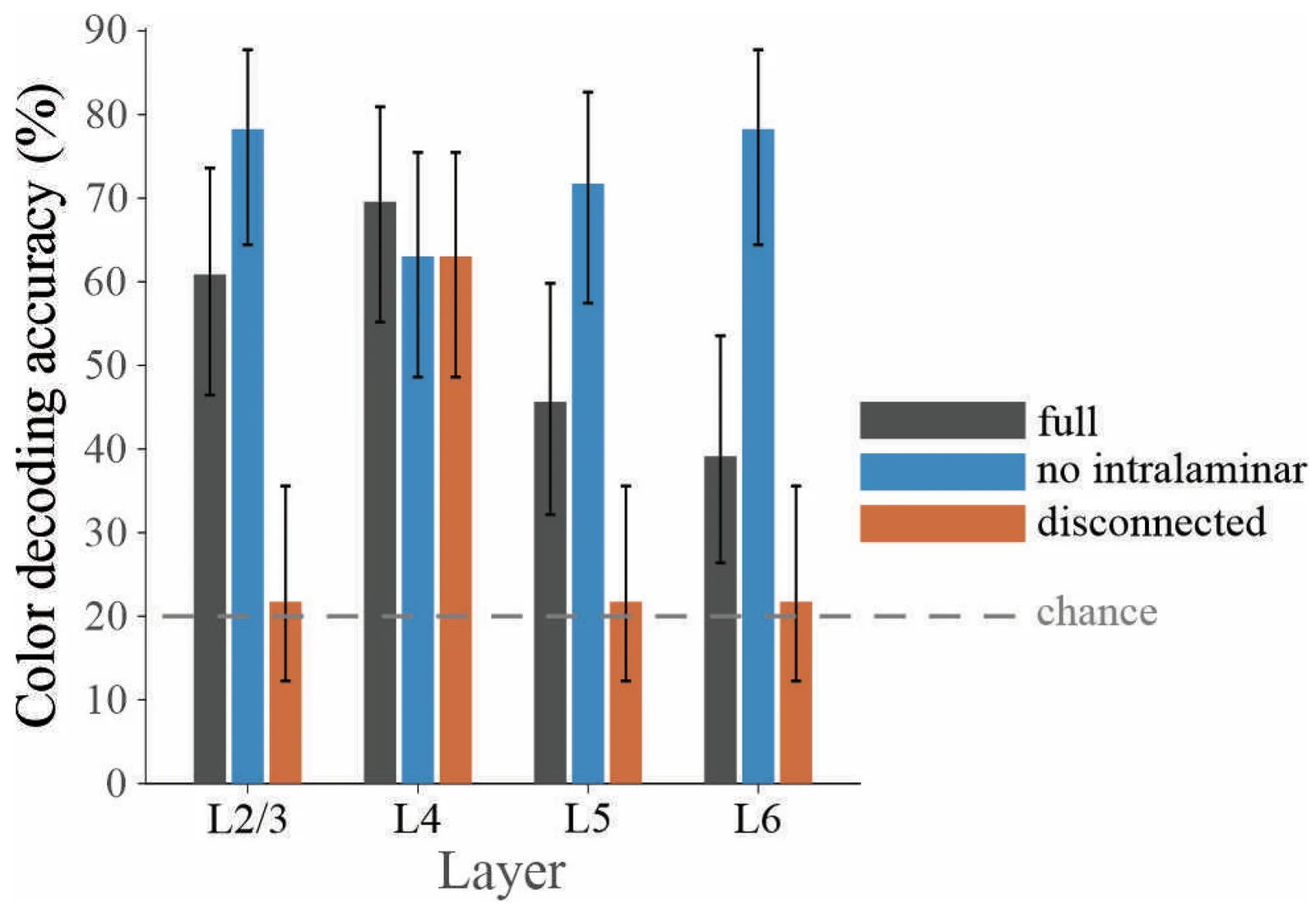
Ablations isolate the contribution of synaptic interaction. Color decoding accuracy per cortical layer for the intact network, for a network with intralaminar recurrence removed (within-layer synapses and gap junctions set to zero, interlaminar pathways intact), and for a fully disconnected network (all synapses and gap junctions set to zero). Neuron models, tonic currents, background activity, stimuli, L4 delivery, features and decoder were identical across the three networks; only the synaptic weight matrices differed. Bars show accuracy with Wilson 95% confidence intervals; dashed line indicates chance (20%). *n* = 46 matched simulations per comparison, paired by color and contrast, at gain 10 µA/cm^2^.

## Data Availability

All data supporting the findings of this study are openly available at https://osf.io/qsypg/overview?view_only=595dbc785d7e436f946928da806f41e5 (accessed on 13 October 2026). Raw HDF5 simulation outputs are available from the corresponding author on reasonable request.

## Abbreviations

AMPA	α-amino-3-hydroxy-5-methyl-4-isoxazolepropionic acid
CI	confidence interval
CV	coefficient of variation
CV_ISI_	coefficient of variation in the interspike interval
f–I	frequency–current (relationship)
GABA	γ-aminobutyric acid
HDF5	Hierarchical Data Format version 5
INT	inhibitory interneuron (connection labels, e.g., PY → INT)
ISI	interspike interval
L2/3, L4, L5, L6	cortical layers 2/3, 4, 5 and 6
NMDA	N-methyl-D-aspartate
NSGA-II	Non-dominated Sorting Genetic Algorithm II
PV	parvalbumin (-expressing interneuron)
PY	pyramidal neuron
SD	standard deviation
SST	somatostatin (-expressing interneuron)
V1	primary visual cortex
VIP	vasoactive intestinal peptide (-expressing interneuron)
